# Modelling the growth, development and yield of *Triticum durum* Desf under the changes of climatic conditions in north-eastern Europe

**DOI:** 10.1038/s41598-021-01273-8

**Published:** 2021-11-05

**Authors:** Kamila S. Bożek, Krystyna Żuk-Gołaszewska, Anna Bochenek, Janusz Gołaszewski, Hazem M. Kalaji

**Affiliations:** 1 Department of Agrotechnology and Agribusiness, Faculty of Agriculture and Forestry, University of Warmia and Mazury in Olsztyn, ul. Oczapowskiego 8, 10-719 Olsztyn, Poland; 2 Center for Bioeconomy and Renewable Energies, University of Warmia and Mazury in Olsztyn, Pl. Łódzki 3, 10-727 Olsztyn, Poland; 3 Department of Genetics, Plant Breeding and Bioresource Engineering, Faculty of Agriculture and Forestry, University of Warmia and Mazury in Olsztyn, Pl. Łódzki 3, 10-724 Olsztyn, Poland; 4 Department of Plant Physiology, Institute of Biology, Warsaw, University of Life Sciences SGGW, Warsaw, Poland

**Keywords:** Agroecology, Computational models

## Abstract

How agricultural ecosystems adapt to climate change is one of the most important issues facing agronomists at the turn of the century. Understanding agricultural ecosystem responses requires assessing the relative shift in climatic constraints on crop production at regional scales such as the temperate zone. In this work we propose an approach to modeling the growth, development and yield of *Triticum durum* Desf. under the climatic conditions of north-eastern Poland. The model implements 13 non-measurable parameters, including climate conditions, agronomic factors, physiological processes, biophysical parameters, yield components and biological yield (latent variables), which are described by 33 measurable predictors as well as grain and straw yield (manifest variables). The agronomic factors latent variable was correlated with nitrogen fertilization and sowing density, and biological yield was correlated with grain yield and straw yield. An analysis of the model parameters revealed that a one unit increase in agronomic factors increased biological yield by 0.575. In turn, biological yield was most effectively determined by climate conditions (score of 60–62) and biophysical parameters (score of 60–67) in the 2nd node detectable stage and at the end of heading. The modeled configuration of latent and manifest variables was responsible for less than 70% of potential biological yield, which indicates that the growth and development of durum wheat in north-eastern Europe can be further optimized to achieve high and stable yields. The proposed model accounts for local climate conditions and physiological processes in plants, and it can be implemented to optimize agronomic practices in the cultivation of durum wheat and, consequently, to expand the area under *T. durum* to regions with a temperate climate.

## Introduction

Durum wheat (*Triticum durum* Desf.) is an important raw material in the food processing industry and the main ingredient in the production of pasta. This wheat species is grown mainly in the Mediterranean Basin, the northern plains of the United States and Canada, the desert areas of south-western USA and northern Mexico, and, to a smaller extent, in other regions of the world^1^.

The annual global production of durum wheat reaches 37 million tons and accounts for 5–6% of the global output of wheat grain^2^. The European Union is the leading producer and consumer of durum wheat. In 2019, the area under *T. durum* in the EU reached 2.2 million ha, including 1.20 million ha in Italy, 0.27 million ha in Spain, 0.27 million ha in France, and 0.29 million ha in Greece^3^. Spring cultivars of durum wheat characterized by high grain quality but lower yields relative to bread wheat are most widely grown. In 2019, the average yield of durum wheat in the EU varied considerably from 2.51 t ha^−1^ in Portugal to 6.21 t ha^−1^ in France^3^. The variations in durum wheat yield are largely attributed to geographic location and climate^4^. In the EU, the highest *T. durum* yields are noted in dry southern regions with high daytime temperatures and low nighttime temperatures. However, the geographic distribution of durum wheat continues to increase due to climate change, and it presently includes selected regions of Central-Eastern Europe^5^. Research indicates that *T. durum* varieties are characterized by higher agronomic performance^1, 6^. In Poland, numerous attempts have been made to introduce durum wheat cultivars grown in southern Europe, but none of them were successful. Therefore, a breeding program was initiated to develop cultivars that are well adapted to the local climate. This study analyzes spring durum wheat cultivar SMH87, one of the selected breeding lines developed during the breeding program.

The cultivated area and yields of durum wheat are determined not only by cultivar, climate conditions and edaphic factors during the growing season, but also by agronomic practices that are synchronized with the plants’ condition in successive stages of growth and development. Durum wheat has specific agronomic requirements, where high temperatures and water deficit are important yield-forming factors as well as stressors that determine the length of each phenological stage. In general, a certain amount of heat has to be accumulated in each phenological stage of wheat^7^, which corresponds to the physiological accumulation of temperature in each growth stage, beginning from tillering, through stem elongation, to heading and grain filling^8^. The hormonal and genetic expression of plants is modified under drought conditions, which shortens the length of each phenological phase and, consequently, affects yields^9, 10^.

The agronomic factors which influence the extent to which the biological potential of durum wheat is harnessed include plant density, nitrogen fertilization and the application of plant growth regulators (PGRs). Durum wheat produces fewer tillers than bread wheat, which is why it has to be more densely sown^11^. Higher plant density in the stand increases the leaf area index (LAI), but it decreases photosynthetic productivity due to mutual shading among individual plants. Carbon dioxide concentration is also higher in dense stands. Extensive tillering increases the number of productive tillers, which contributes to higher values of the harvest index^12^. The plant yield which is the outcome of physiological processes and chlorophyll concentration in photosynthetic organs is closely related to nitrogen fertilization^12–16^. Nitrogen enhances leaf greenness which is expressed by chlorophyll content, plant vitality, increase in fresh biomass and yield formation in the generative growth stages of spring durum wheat^17, 18^. However, higher nitrogen rates can decrease plant resistance to lodging^19^. The model developed by Berry and Spink^20^ predicted that severe lodging up to 90° from the vertical plane can reduce yields by approximately 61%.

At present, PGRs are applied mainly in intensive cereal production to shorten stems and decrease susceptibility to lodging^21–23^. Lodging occurs before flowering and in the early grain filling stage, and it can significantly reduce yields by disrupting light absorption in the stand, decreasing water availability, and compromising the transport and translocation of nutrients and photosynthetic products. High temperature combined with high precipitation can lead to the development of saprophytic fungi in wet stands during grain ripening, and it can increase the risk of kernel germination before harvest. Lodging also increases the time and energy required for grain harvesting and drying, which raises production costs. Therefore, the application of PGRs contributes to increasing yields, improving grain quality and increasing profitability in agricultural production^22, 24^.

Agronomic factors influence the photosynthetic rate, transpiration rate, water use efficiency (WUE) and chlorophyll content which affect durum wheat yields and yield components^17, 25–28^. Conventional evaluations of photosynthetic activity and transpiration rates in cereals are based on measurements of the photosynthetic organs, i.e., the flag leaf, the leaves below the flag leaf, and the ear. During water deficit, the net photosynthetic rate decreased to a lesser extent in durum wheat ears than in flag leaves^25^. In turn, Bousba et al.^27^ and Prakash and Ramachandran^29^ argued that a decrease in photosynthetic rate could result from lower chlorophyll content.

Physiological analyses of plants grown under specific field conditions support the identification of biotic and abiotic stressors and promote rational decision-making in agronomic practice concerning, for example, the selection of drought-tolerant genotypes^30^. According to Abbad et al.^25^, the photosynthetic process in durum wheat is closely related to water availability in each stage of plant growth and development and during the entire growing season. It was demonstrated that the net photosynthetic rate was higher in flag leaves than in spikes under drought conditions. Optimal agronomic practices can increase photosynthetic productivity during plant growth and development by prolonging active photosynthesis. Certain cultivation measures can be taken to delay leaf senescence. Thomas and Howarth^31^ demonstrated that carbon accumulation in plants increased by around 11% when leaf senescence was delayed by two days. This observation has important implications for the production of crops at high temperatures which accelerate senescence and inhibit the translocation of assimilates to grain.

Numerous attempts have been made to model the impact of climatic, agronomic and physiological variables on wheat yields^32–37^. The developed models support comprehensive characterization of the growth and development of wheat plants, and they promote decision-making in crop production under specific soil and climate conditions. Orlando et al.^38^ were described several useful models, including CERES-wheat, SWHEAT, AFRCWHEAT2 and APSIM-N-wheat. The authors argued that variations in the yield and quality of durum wheat are geographically conditioned, and they observed that durum wheat producers in Eastern Europe have a low competitive advantage. Intensive production of durum wheat has started relatively recently in the north-eastern part of the European Union. Therefore, there are no published studies investigating the growth and development of *T. durum* under the agronomic, soil and climatic conditions of north-eastern Europe with the use of the existing models, and new original modeling approaches have not been proposed to date.

The aim of this study was to: (i) analyze the climatic, agronomic and physiological conditions associated with the growth and development of durum wheat and the possibility of expanding the area under *T. durum* to north-eastern Europe in the context of ongoing climate change, and (ii) model the growth, development and yield of durum wheat.

## Results

### Climatic conditions and phenology

The growth and development of *T. durum* plants was moderately differentiated by weather conditions in the analyzed years (Table 1).

**Table 1:** The duration of growing seasons (Days), sum of temperatures (Temp.) and sum of precipitation (Prec.) during the growth and development of *T. durum* Desf. in the analyzed years.

	2015			2016			2017		
	Days	Temp. (°C)	Prec. (mm)	Days	Temp. (°C)	Prec. (mm)	Days	Temp. (°C)	Prec. (mm)
Growing season (total)	136	2011.3	366.7	132	1985.6	360.1	145	2068.9	350.5

**Figure 1:**
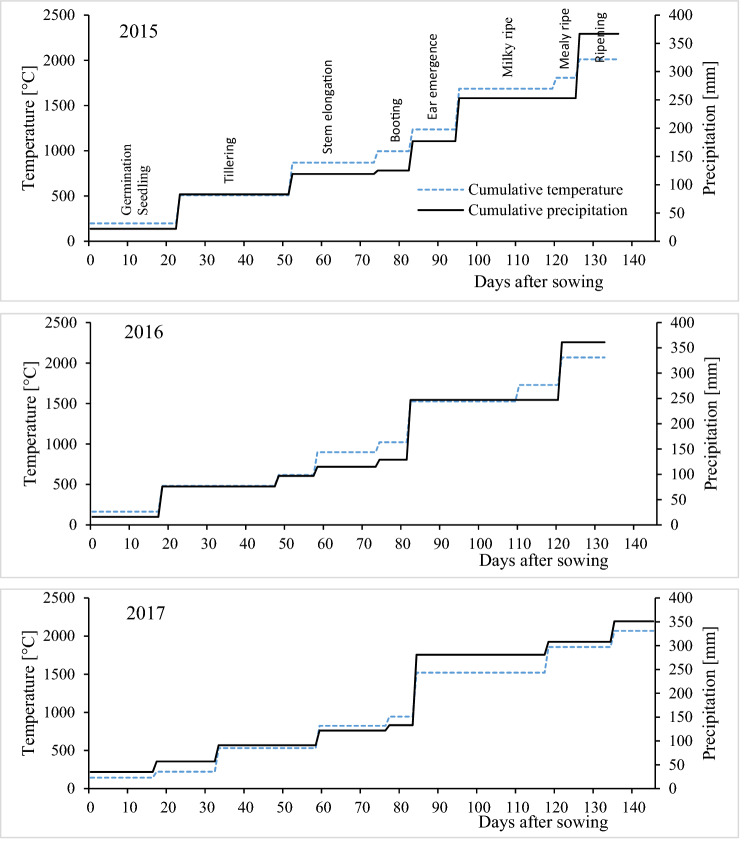
Cumulative temperatures and precipitation in the phenological phases of *T. durum* in 2015–2017.

The growing seasons of 2015, 2016 and 2017 lasted 136, 132 and 145 days, respectively; the sum of temperatures was determined at 2011.3, 1895.6 and 2069.9 °C, respectively, and the sum of precipitation was determined at 366.7, 360.1 and 350.5 mm, respectively. However, a comparison of cumulative temperatures and precipitation in the phenological phases of *T. durum* in each year of the study indicates that temperature and precipitation could have influenced the duration of the examined phases and plant growth indicators (Fig. 1). Weather conditions were generally favorable for the growth and development of *T. durum* in 2015 and 2016. Cumulative temperatures and precipitation were quite similar in 2015 and 2016 up to the booting stage, but precipitation levels in successive stages were higher in 2016 than in 2015. The growing season was shortest in 2016 and longest in 2017, mainly due to low temperatures during sowing and seed germination, and high precipitation during tillering, grain formation and ripening.

### Biophysical parameters: LAI and SPAD

The LAI denotes the area of photosynthetic tissue per unit ground surface area (m^2^ m^−2^). The LAI is directly associated with plant canopy, and it is an indicator of net primary production, water and nutrient use, and the carbon balance. SPAD is a measure of leaf greenness that is directly associated with chlorophyll content and nitrogen sufficiency.

The main effects of LAI and SPAD were analyzed separately in the framework of the Zadoks scale to reveal the significant effects of years, nitrogen rates and sowing density, and an absence of significant effects associated with the application of the growth regulator (see Tables 1.1–1.6 in the Supplementary Information). In the analyzed years, LAI and SPAD were similar in the 2nd node detectable stage (Z32), but they differed in the stem elongation stage (Z45) and the ear emergence and heading stage (Z59), when LAI values were higher and leaf greenness values were lower in 2016 and 2017 than in 2015. These findings can be attributed to moderate temperatures and precipitation in 2015, and high precipitation in the critical growth stages in the remaining years. The general trend associated with the nitrogen rate was similar across the examined growth stages, i.e. a significant increase in LAI and SPAD values with nearly identical effects were noted in treatments with nitrogen rates of 80 and 120 kg ha^−1^. A similar trend was observed in sowing density. In treatments with a sowing density of 450 and 550 germinating seeds per m^2^, LAI values continued to increase, whereas SPAD values were below those noted in the treatment with a sowing density of 350 germinating seeds per m^2^. The only significant interaction was observed between years and nitrogen rates.

The average values of LAI continued to increase in successive growth stages and were determined at 1.30 at Z32, 1.75 at Z45, and 1.99 at Z59. In turn, leaf greenness was significantly lower in the stem elongation stage (Z45) than in the preceding (Z32) and subsequent (Z59) stages.

The significant effect of the *years* × *growth stages* interaction for LAI and SPAD values resulted from similar means in stage Z32 in all years, as well as higher LAI values and lower SPAD values in subsequent growth stages in 2016 and 2017 than in 2015. In 2015, the increase in the nitrogen rate induced only a rising trend in LAI and SPAD values, whereas significant differences were observed in 2016 and 2017. To summarize, it should be noted that in successive Zadoks growth stages, the interactions between years, nitrogen rates and sowing density exerted significant effects on LAI and SPAD values, whereas the effects of *year* × *nitrogen rate* interactions were significant only in selected growth stages.

### Contribution of different sources of variation to physiological and biophysical parameters of plant growth

The calculated eta-squares η^2^ provide information about the contribution of different sources of variation to physiological variables (Table 2). The experimental years and agronomic factors (33.1% and 38.6%), growth stages, and interactions with other factors (32.5% and 39.3%) and random factors (34.4% and 22.1%) made similar contributions to the variation in the LAI and chlorophyll content. The variation in the net photosynthetic rate was related mostly to variations across years (32.6%) and the interactions between growth stages and other factors (24.3%). The variation in the transpiration rate was attributed mostly to the main effects of growth stages (45.8%) and the *year* × *growth stage* interaction (16.1%). Instantaneous WUE was strongly determined by variation in agronomic factors and growth stages (22.3% and 21.1%, respectively).

**Table 2:** Eta-square (η^2^) values for the sources of variation in the leaf area index (LAI), chlorophyll content (SPAD), net photosynthetic ratio (Pn), transpiration rate (E) and instantaneous water use efficiency (WUE).

Source of variation	LAI	SPAD	Pn	E	WUE
Years Y	2.9	23.0	32.6	2.7	2.9
Agronomic factors A	22.3	11.8	0.4	2.0	22.3
Y × A	6.6	3.3	3.2	2.7	6.6
A × A	1.3	0.5	1.0	0.5	1.3
Total Y&A	33.1	38.6	37.2	7.9	33.1
Growth stage G	21.1	15.6	7.0	45.8	21.1
Y × G	9.4	21.5	15.9	16.1	9.4
A × G	2.1	2.1	1.4	2.3	2.1
Total G	32.5	39.3	24.3	64.2	32.5
Random factors	34.4	22.1	38.5	27.9	34.4

It is worth noting that the variation in agronomic factors made a considerable contribution to the total variation in LAI (22.3%) and SPAD (11.8%), but only a marginal contribution to the net photosynthetic rate (0.4%) and transpiration (2.0%).

### Photosynthetic indicators— net photosynthetic rate, transpiration rate, and instantaneous water use efficiency

The effects of the net photosynthetic rate (Pn), transpiration rate (E) and instantaneous WUE were highly differentiated in successive growth stages, and relatively small differences were noted for agronomic factors (see Tables 2.1–2.9 in the Supplementary Information). At the same time, the analyzed photosynthetic indicators differed in successive stages of growth. The net photosynthetic rate was similar in the 2nd node detectable stage (Z32) and the stem elongation stage (Z45) at 29.7 μmol CO_2_ m^–2^ s^–1^, and it was 15% higher at the end of the heading stage (Z59) than in the preceding stages. The transpiration rate continued to increase by 60% on average in successive stages of growth and development, from 1.59 H_2_O m^–2^ s^–1^ in stage Z32, to 2.52 mmol H_2_O m^–2^ s^–1^ in stage Z45, and 4.06 mmol H_2_O m^–2^ s^–1^ in stage Z59.

**Figure 2:**
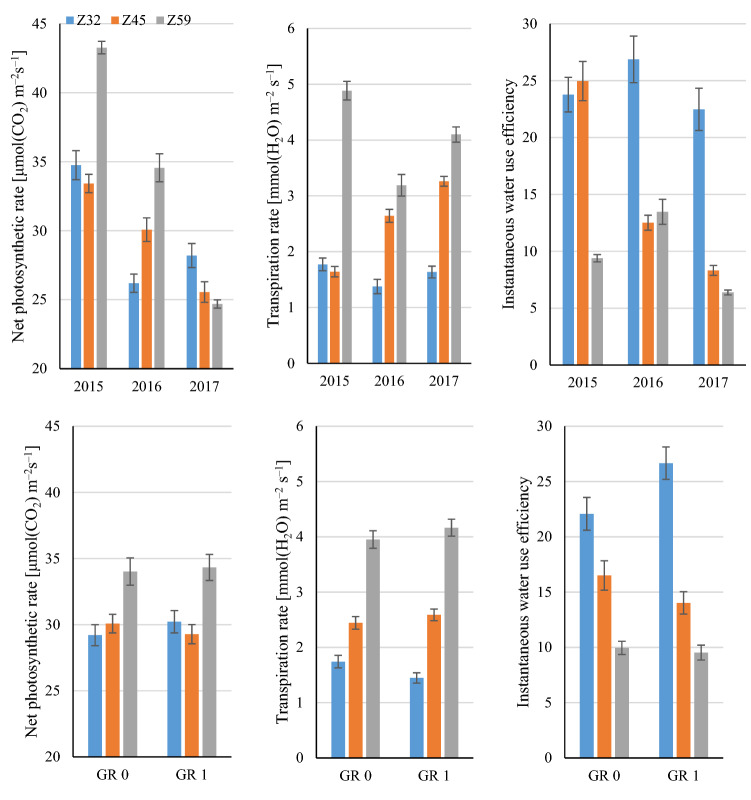
Mean values and standard error of photosynthesis indicators for *year* × *growth stage* (upper) and *growth regulator* × *growth stage* interactions (GR 0—without growth regulator, GR 1—with growth regulator).

An analysis of the results noted in different growth stages across years revealed significant *year* × *growth stage* and *growth regulator* × *growth stage* interactions (Fig. 2).

The *year* × *growth stage* interaction resulted from differences in the rates of photosynthesis and transpiration in the analyzed growth stages across years. In 2015, the net photosynthetic rate was similar in the first two growth stages, and it increased by around 30% at the end of the heading stage (Z59). In 2016, the photosynthetic rate continued to increase in successive growth stages. In 2017, the net photosynthetic rate was around 10% higher in the 2nd node detectable stage (Z32) than in the stem elongation stage (Z45) and at the end of the heading stage (Z59). The transpiration rate increased significantly in successive stages of plant growth and development, and the only exception was noted in 2015, when the analyzed parameter was similar in stages Z32 and Z45. The WUE index was highest in stage Z32, and a significant interaction was noted due to the correlation between the net photosynthetic rate and the transpiration rate in the remaining stages. Water use efficiency was similar in stages Z32 and Z45 in 2015, and in stages Z45 and Z59 in 2016, whereas significantly lower values in successive stages of plant growth were noted in 2017.

The growth regulator was the only agronomic factor that induced significant differences in the net photosynthetic rate across the examined growth stages. Photosynthesis indicators were similar regardless of the application of the growth regulator, and significant interactions resulted mainly from varied disproportions between the end of heading and the stem elongation stage in treatments with and without the application of the growth regulator.

It should be noted that the interactions between growth stages and nitrogen rates and sowing density were not significant, which implies that the effects of the interactions between increasing nitrogen rates and sowing density on photosynthetic indicators in successive growth stages were similar to the average values of photosynthetic indicators in the corresponding growth stages (Supplementary Information).

### Agronomic traits

The means for yield components and yield are presented in Tables 3.1–3.8 of the Supplementary Information. Stem length was differentiated by the nitrogen rate and *nitrogen rate* × *year* interaction. Nitrogen rates of 80 and 120 kg N per ha increased stem length by 11% and 13%, respectively, relative to the unfertilized control. The significant *year* × *nitrogen rate* interaction resulted from the fact that the nitrogen-induced increase in stem length was smaller in 2015 (0.07 cm per 1 kg of nitrogen) than in 2016 and 2017 (0.09 cm per 1 kg of nitrogen). In 2015, ear length was similar to that noted in the remaining years, and only in 2017, ear length was 7% higher than in 2016. Ear length and the number of kernels per ear increased with a rise in nitrogen rate and decreased with a rise in sowing density.

Grain weight per ear and 1000 kernel weight were highest in 2015 and significantly lower in the following years. Grain weight per ear increased only in response to the nitrogen rate of 120 kg ha^−1^, but 1000 kernel weight was not affected. Both traits decreased with a rise in sowing density. The significant *year* × *nitrogen rate* and *year* × *sowing density* interactions for both traits can be largely attributed to the magnitude of differences between years, rather than an increase or a decrease in this trend.

The biological yield (grain and straw) differed across years and nitrogen rates. In 2016, the biological yield was similar to that noted in 2015 and significantly higher (by 30%) than that noted in 2017. The biological yield increased by 28% and 35% in response to nitrogen rates of 80 and 120 kg ha^−1^, respectively, relative to the unfertilized control. The significant *year* × *nitrogen rate* interaction was associated with variations in nitrogen use efficiency, and the difference between maximal biological yield was determined at 0.5 t ha^−1^ in 2015, 2.3 t ha^−1^ in 2016, and 2.8 t ha^−1^ in 2017.

Grain yield was similar in 2015 (4.94 t ha^−1^) and 2016 (5.38 t ha^−1^), and it was significantly lowest in 2017 (3.87 t ha^−1^). Straw yield was highest in 2016 (2.86 t ha^−1^), and it exceeded the values noted in the remaining years by 16%. The harvest index was similar in 2015 and 2016, and it was 9% lower in 2017. Grain yield increased by 30% and 36%, whereas straw yield increased by 20% and 35% in response to the nitrogen rates of 80 and 120 kg ha^−1^, respectively. A minor increase in grain yield (3%) was observed in treatments with a sowing density of 550 seeds m^−2^ relative to the remaining sowing densities.

### Path modelling

A simple correlation analysis of manifest variables in all phenological stages revealed significant correlations between the LAI and leaf greenness (SPAD) only in stage Z32, as well as a very strong correlation between the net photosynthetic rate and the transpiration rate, which was positive in stages Z32 and Z45 and negative in stage Z59. No simple correlations were noted between the indicators of physiological processes (Pn, E, WUE) and biophysical parameters (LAI, SPAD). AAll correlations between the manifest variables of yield components and biological yield were statistically significant, excluding the correlation between stem length and ear length (Supplementary information).All correlations between the manifest variables of yield components and biological yield were statistically significant, excluding the correlation between stem length and ear length (Supplementary Information). The outer and inner PLS-PM models well fit the data, and their goodness of fit was determined at 0.973 and 0.786, respectively. The outer weights provide information about the relative importance of a manifest variable for the corresponding latent variable (for details please see the Supplementary Information). Outer weights that exceed 0.3 are considered meaningful. By the same token, loading estimates represent the correlations between a latent variable and the corresponding manifest variables. Loadings higher than 0.7 capture more than 50% of the variability contributed by a latent variable to the corresponding manifest variable. In general, both indicators in the outer model, i.e. outer weights and loadings, exceeded the thresholds, which indicates that manifest variables were strongly related with latent variables. Growth regulators (${w}_{GR}$ = − 0.007) and the length of the growing season (${w}_{DAYS}$=0.197) provided the only evidence for the low explanatory value of latent variable A (agronomic factors).

In the inner model, all equations that regressed latent variables well fit the data and were statistically significant (Table 3). The latent variables expressed by the value of *R*^2^ increased in successive stages of *T. durum* growth and development, from 0.218 in physiological processes in stage Z32 (Table 3, Eq. 1) to 0.698 and 0.708 in yield components and Biological Yield, respectively (Table 3, Eqs. 7 and 8). It is worth noting that in successive stages of growth, the value of physiological processes was relatively lower in comparison with biophysical parameters.

**Table 3:** Parameters of regression models for latent variables.

No	Inner model	Parameter	Value	Pr >|t|
1	PP32 = *β*_1_*A + *β*_2_*CC32	*β*_1_	0.464	0.000
		β_2_	− 0.048	0.699
		*R*^2^	0.218	
2	BP32 = *β*_1_*A + *β*_2_*CC32 + *β*_3_*PP32	*β*_1_	0.851	0.000
		β_2_	0.197	0.030
		*β*_3_	− 0.382	0.000
		*R*^2^	0.614	
3	PP45 = *β*_1_*A + *β*_2_*CC45	*β*_1_	0.515	0.000
		β_2_	0.171	0.153
		R^2^	0.294	
4	BP45 = *β*_1_*A + *β*_2_*CC45 + *β*_3_*PP45	*β*_1_	− 0.052	0.588
		*β*_2_	0.826	0.000
		β_3_	− 0.065	0.499
		*R*^2^	0.674	
5	PP59 = *β*_1_*A + *β*_2_*CC59	*β*_1_	0.550	0.000
		β_2_	0.191	0.099
		R^2^	0.339	
6	BP59 = *β*_1_*A + *β*_2_*CC59 + *β*_2_*PP59	*β*_1_	0.720	0.000
		*β*_2_	0.542	0.000
		β_3_	− 0.395	0.001
		*R*^2^	0.573	
7	YC = *β*_1_*A + *β*_2_*BP32 + *β*_3_*BP45 + *β*_4_*BP59 + *β*_5_*CC	*β*_1_	− 0.304	0.008
		*β*_2_	0.482	0.004
		*β*_3_	0.011	0.897
		β_4_	0.556	0.001
		β_5_	− 0.171	0.144
		*R*^2^	0.698	
8	BY = *β*_1_*A + *β*_2_*CC + *β*_3_*YC	*β*_1_	0.448	0.000
		β_2_	− 0.352	0.000
		β_3_	0.422	0.000
		*R*^2^	0.708	

*Refer to Table 1 for the legend.

The analysis of path coefficients (*β*_i_) revealed that agronomic factors (A) and climate conditions (CC) in stages Z32, Z45 and Z59 exerted a specific influence on physiological processes (PP) and biophysical parameters (BP) of *T. durum* plants. Agronomic factors directly determined physiological processes in all stages and biophysical parameters in stages Z32 and Z59. At the same time, climate conditions did not exert a direct influence on physiological processes in any stage, but directly affected biophysical parameters in all stages. All of the modeled parameters, i.e. agronomic factors, climate conditions and physiological processes, significantly influenced biophysical parameters in stages Z32 and Z59, but not Z45. Consequently, it can be stated that agronomic factors were the main determinant of variability in physiological processes (photosynthesis, transpiration) in a model evaluating the impact of agricultural practices on yield and the manifest variables associated with *T. durum* growth and development. At the same time, physiological processes made a significant but negative contribution to biophysical parameters. A one unit increase in photosynthesis processes with constant values of agronomic factors and climate conditions implies a decrease of − 0.382, − 0.065 and − 0.395 in biophysical parameters in stages Z32, Z45 and Z59, respectively.

**Figure 3:**
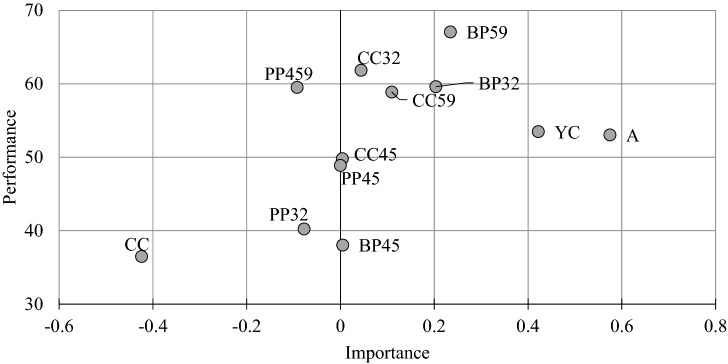
Importance-Performance Map Analysis presenting the impact of latent variables on biological yield (A—agronomic factors, YC—yield components, CC32, CC45, CC59—climate conditions in growth stages, PP32, PP45, PP59—physiological processes, BP32, BP45, BP59—biophysical parameters in the phenological stages of plant growth and development Z32, Z45 and Z59, CC—climate conditions for the entire growing season).

The performance of every preceding latent variable in terms of its total impact on the target latent variable, i.e. the biological yield of *T. durum* (IPMA – Importance-Performance Map Analysis), was analyzed to highlight latent variables associated with agricultural practices that improve biological yield. The total effect (importance) of preceding latent variables (A, CC32, PP32, BP32, CC45, PP45, BP45, CC59, PP59, BP59, YC and CC) on the anticipated performance of the specific target (Biological Yield) is presented in Fig. 3.

The importance and performance of latent variables that influenced the biological yield of *T. durum* varied. The biological yield of *T. durum* was affected mostly by agronomic factors (A), followed by yield components (YC) and biophysical parameters (BP) in growth stages Z59 (BP59) and Z32 (BP32), climate conditions in stage Z59 (CC59), and climate conditions in stage Z32 (CC32). A one unit increase in the above latent variables led to an increase of 0.575, 0.422, 0.234, 0.203 and 0.109 units in biological yield, respectively. At the same time, the performance scores of these latent variables were determined at 53.1, 53.5, 67.1, 59.6 and 61.8, respectively (scores closer to 100 denote higher performance). The remaining latent variables, in particular climate conditions for the entire growing season and physiological processes in stage Z32, were characterized by low importance and exerted a relatively small effect on biological yield performance.

The results of the importance-performance analysis clearly indicate that latent variables have considerable potential to optimize the agricultural conditions for the growth and development of *T. durum* plants.

## Discussion

Climate change and global warming have different implications for crop production. Due to the projected increase in temperature and decrease in water precipitation, the range of species traditionally grown in southern Europe may expand to the northeastern part of the continent. The length of the growing season in northern Europe is also expected to increase^39^. In the present study, the growing season spanned 132 to 145 days (depending on weather conditions in a given year) and was longer than in typical wheat growing areas where daily temperatures exceed 32 °C (Mauritania, Senegal) and the growing season lasts only 92 days^40^.

Air temperatures during the growing season were similar to those estimated by Bozzini^41^ for southern Europe, where the optimal temperature for the growth and development of durum wheat is 15 °C. Grain filling is a phenological phase of critical importance for grain quality. In the grain filling stage, mean daily temperature was determined at 19–20 °C, and mean daily precipitation reached 0–10 mm. These temperatures are significantly below the maximum values (35 °C) that can be tolerated by durum wheat. According to Blum^42^ and Blumenthal et al.^43^, high temperatures in the grain filling stage can improve flour and dough quality. A long-term study of spring wheat demonstrated that wheat is most sensitive to changes in temperature between tillering and heading stages and between heading and dough stages, when a temperature rise of 1 °C increased grain yields from 208 to 554 kg ha^−1^^44^.

Water deficit is an important determinant of crop yield and quality^45, 46^. Durum wheat genotypes that are stable in high yielding across different environments is difficult to obtain due to high genotype × environment interaction. Therefore, it is considered that selection of traits determining high yielding under water stress can be decisive in effective durum wheat genotype selection^47–49^. Boudjabi et al.^49^ reported that water stress affects durum wheat development by reducing leaf area, plant biomass and consequently grain yield. According to De Jong et al.^50^ the low rainfall can have a more negative impact on durum wheat yields than soil water deficits, especially in semi-arid regions. The water requirements of durum wheat during the growing season were estimated at 350–450 mm^51^. In the present study, precipitation approximated the lower limit of the above range. Rainfall before sowing contributes to the yield potential of crops. In this period, a 1 mm difference relative to the long-term average resulted in yield fluctuations of 44–157 kg ha^−1^, depending on the soil quality class. In turn, precipitation between emergence and tillering stages and between heading and dough stages did not exert such an effect, leading to yield fluctuations of 2 to 40 kg grain ha^−1^, subject to the soil quality class^44^.

In a study by Cabrera-Bosquet et al.^52^, nitrogen significantly enhanced plant growth and development by increasing above-ground biomass and the LAI measured after anthesis on the flag leaf. The above parameters decreased between the booting stage and the watery ripe stage. In the current study, durum wheat plants responded similarly to nitrogen fertilization in different stages of development. It suggests that nitrogen doses differentiated plant growth and development indicators during the whole season. In comparison with the unfertilized treatment, LAI and SPAD values increased already in response to the nitrogen rate of 80 kg N ha^−1^, but a further increase in the nitrogen rate did not induce significant changes in these parameters. Regardless of the nitrogen rate, the net photosynthetic rate, the transpiration rate and WUE were similar, excluding the transpiration rate in stage Z45 which decreased in response to 120 kg N ha^−1^ in comparison with lower nitrogen rates. The biophysical parameters were also modified by sowing density. The LAI continued to increase with a rise in sowing density to 450 and 550 germinating seeds per m^2^, whereas SPAD decreased below the values noted at the density of 350 germinating seeds per m^2^. The above increase did not result in significant changes in photosynthetic indicators in the different treatments. These observations suggest that agronomic factors may exert a stronger influence on biophysical parameters (LAI, SPAD) of *T. durum* than physiological parameters (Pn, E, WUE). At the same time, variations in both groups of parameters were strongly influenced by agronomic factors at different growth stages. These observations are confirmed by the absence of correlations between biophysical and physiological parameters. The positive correlation between LAI and SPAD and the negative correlation between net photosynthetic rate and transpiration rate indicate that these parameters can be used in the evaluation of agronomic practices at the final stages of plant development.

In a study by Allahverdiyev et al.^53^, flag leaf area was strongly correlated with plant height, ear weight, grain number per ear, and grain weight per ear, suggesting that large leaf area contributes to the formation of more assimilates transported to the ear. Chlorophyll content, in turn, was positively and significantly correlated with plant height, ears per m^2^, ear length, aboveground biomass, plant weight, grain yield, ear weight, ear number per ear and grain number per ear. Grain weight per ear was also associated by positive and significant correlation with most of the physiological parameters.

In crop plants, photosynthesis is the primary source of dry matter production and grain yield^54^. Leaf photosynthesis can vary with leaf age, position, leaf area, and growth stage. Leaf gas exchange is also affected by daily changes in weather, including light intensity, temperature, and relative humidity^55^. In the present study, net photosynthetic rate (Pn) was determined by climatic and edaphic conditions across years and growth stages, but it was not strongly influenced by agronomic factors. This can probably be attributed to the fact that weather conditions, mostly moderate rainfall, were generally favorable for plant growth in the experimental years. According to Pinthus^56^, the high rainfall during growth and high nitrogen applications can promote stem elongation and increase the risk of lodging. Yield losses due to lodging can be predicted based on reduced photosynthetic activity resulting from changes in stand architecture^20, 25^.

A decrease in photosynthetic rate could result from lower chlorophyll content^27, 29^. Such trend was observed in the present study but the correlation between the photosynthetic rate and SPAD was not significant. Abbad et al.^25^ found that a decrease in photosynthetic rate of flag leaf and ear was correlated with a decrease in transpiration rate. A study analyzing the effects of water stress on *T. durum* showed a positive but not significant relationship between pH and chlorophyll content^30^. The cited study also showed that leaf gas exchange parameters (Pn and E) were positively correlated with days to shooting, plant height, aboveground biomass, ear weight, ear width and number of spikelets per ear. In turn, Bogale et al.^57^ was reported the absence of significant correlations between Pn and grain yield and argued that selection for higher rates of leaf photosynthesis does not increase yield, probably because the source is less limiting than the sink. In the current study, the nitrogen rate of 80 kg ha^−1^ exerted the greatest influence on both yield and yield components.

Soil nitrogen availability increased with an increase in nitrogen rate of 80 and 120 kg N ha^−1^, which significantly increased grain yield by 30% and 36% (to peak yield), respectively, and grain yield stabilized at 5.3 t ha^−1^ with adequate nitrogen availability. Similar relationships between nitrogen and wheat grain yield have been noted by many authors^13, 14, 16, 58, 59^. The relationship between nitrogen fertilization and durum wheat yield was modeled by linear regression, where biological yield was 43% higher and grain yield was 94% higher in fertilized treatments than in the unfertilized control^14^. According to Pinhtus^56^, excess nitrogen and the interaction between nitrogen and high rainfall during the growing season increase the risk of lodging and contribute to significant yield reduction.

The present results on the impact of nitrogen fertilization on yield components and yield coincide with those reported by Cabrera-Bosquet et al.^52^ who found that nitrogen significantly affected grain yield, grain weight per plant, number of ears per plant and number of grains per ear. However, nitrogen fertilization had no significant effect on harvest index (HI) or 1000-grain weight. Probably, the increased nitrogen doses are accompanied with the increase of both grain and straw yield at the similar ratio of grain yield to straw yield around 1.8.

During the growing season, nitrogen is distributed from older to younger leaves, which decreases the greenness of older leaves. Variations in the leaf greenness index were not observed in the 2nd node detectable stage (Z32) and the stem elongation stage (Z45) when SPAD values were lowest. Nitrogen induced a significant increase in leaf greenness, but this parameter was identical in treatments supplied with 80 kg N ha^−1^ and 120 kg N ha^−1^. The noted SPAD values confirm the previous observation that grain yield stabilized at a maximum level in response to 80–120 kg N ha^−1^.

Sowing density is closely correlated with LAI. A linear increase in LAI was observed when sowing density increased from 350 to 450 and 550 seeds m^−2^. The LAI increased by around 10% with an increase in stand density, in particular in stages Z32 and Z59, which promoted light interception and decreased the quality of light available to weeds. According to Beres et al.^60^, the higher plant density can increase the competitive ability of wheat plants against weeds, thus increasing grain yields. In the present study, sowing density did not influence plant height, but it affected all yield components that were maximized at the highest sowing density of 550 seeds m^−2^, and grain yields in treatments with a sowing density of 450 seeds m^−2^ were in between the values noted in treatments with the lowest and highest sowing densities. These findings indicate that an increase in sowing density did not affect biological yield, but it influenced the grain yield to straw yield ratio. The yield-forming effects of higher sowing density in durum wheat were confirmed by Naseri et al.^11^, where the highest number of 348 spikes m^−2^, 34.4 grains per spike, and the highest grain yield of 4.22 t ha^−1^ were noted at a sowing density of 400 seeds m^−2^. At this sowing density, the HI reached 0.486, and it was considerably lower than in the present study. In the work of Panahyan-e-Kivi et al.^61^, higher sowing density increased all of the analyzed plant parameters. Grain yields peaked at 3.69–4.62 t ha^−1^ in response to the sowing density of 400 seeds m^−2^ and the nitrogen rate of 140 kg N ha^−1^, and the HI index ranged from 0.427 to 0.522. In the current study, the recommended sowing rate of 450–550 seeds m^−2^ is approximately 25% higher than that suggested in the literature. Isidro-Sánchez et al.^62^ reported the highest grain yield at a sowing density of 272–380 seeds m^−2^, whereas in the work of Nilsen et al.^63^, a linear increase in grain yield was noted when sowing density was increased from 150 to 450 seeds m^−2^.

In the present study, weather conditions significantly differentiated durum wheat growth however lodging was not observed and application of growth regulator had no significant main effects on plant traits. However, an evaluation of the physiological parameters of the plants showed significant differences in the interaction effects of the growth regulator across the growth stages and years of the study, mainly resulting from the differences between the end of shoot and stalk elongation stages in the treatments with and without the application of the growth regulator.

Many wheat growth and development models reliably predict grain yields, and models that are strictly user-oriented provide support for crop management, risk analysis, and strategic planning at local and global scales, including in the context of environmental processes such as climate change^32, 34, 36, 38, 64^. Durum wheat yield prediction models generally rely on the linear relationship between the measured predictors and the dependent variable. The model proposed in the present study complements such approach by classifying the measurable predictors with the highest predictive power into groups of latent variables reflecting different aspects of durum wheat crop growth and development. The model PLS-PM describes the extent to which biological yield was determined by climate conditions at different phenological stages and throughout the growing season, agronomic factors, biophysical parameters and physiological processes at critical stages of plant development and yield components. Agronomic factors (nitrogen fertilization, seeding density, and growth regulator application) determined photosynthetic and transpiration rates (physiological processes) and differentiated leaf greenness and leaf area (biophysical parameters), but only at the second node detection stage (Z32) and at the end of heading (Z59).

To conclude the above discussion, three aspects of model usability are to be taken into account—variability, results and applications.

*Variability* Climate change symptoms in Central-Eastern Europe, such as a rise in mean temperature and a fall in total precipitation during the growing season, as well as projections of potential global climate change, such as a higher incidence of heat waves, a decrease in summer precipitation levels, and greater variability in crop yields, as well as success in breeding new varieties of durum wheat, all point to increased variability in crop yields.

*Results of modeling* The model proposed in the study fits well with empirical data describing durum wheat yields under soil and climatic conditions of northeastern Poland. The model showed that agronomic factors was the most important latent variable, with a one unit increase in agronomic factors increasing the variable biological yield by 0.575. An analysis of the extent to which latent variables influenced biological yield (performance) again revealed that biological yield was most effectively determined by climate conditions and biophysical parameters at the 2nd identifiable stage of the node and at the end of the head. The configuration of latent and manifest variables in the model was responsible for less than 70% of the potential biological yield, implying that the growth and development of durum wheat in north-eastern Europe can be optimized to provide high and stable yields.

*Model application* The proposed model, partial least square path modeling, can be implemented to increase production, promote good agricultural practices, and ultimately expand durum wheat acreage in temperate zone. The results of this study suggest that weather variability significantly modifies physiological processes in plants and yield components, contributing to the risk of unstable durum wheat yields over years.

## Methods

All methods of the paper were carried out in accordance with relevant guidelines and regulations.

### Material

The material for the study was an original spring cultivar of durum wheat SMH 87. According to the Plant Breeding Station in Smolice^65^ which conducts research into Polish cultivars of durum wheat, SMH 87 is an awned and short-stemmed cultivar with a height of 80–85 cm, which is resistant to lodging and enters the heading stage two days earlier on average than bread wheat. The analyzed cultivar has a 1000 kernel weight of 51–54 g. Its grain is characterized by high protein content (15.8%), considerable hardness (188 BU) and high content of pigments (5.7 ppm). For collection of seeds or plants of *T. durum* Desf no relevant permits or permissions were needed. In every study year (2015, 2016, 2017) the original sowing material of *T. durum* Desf (cultivar SMH 87) was bought directly from SHR Smolice. According to the letter from the CEO of SHR Smolice, in Poland, no special permits for collection of seeds or plants of registered cultivars *T. durum* Desf are required for both scientific research and cultivation in production fields.

### Field experiment

Durum wheat cv. SMH87 was grown in a field experiment in the Agricultural Experiment Station in Bałcyny, Poland (53°40′ N, 19°50′ E) in 2015–2017. In the context of climate change the three consecutive years of study composed the sample of differentiated climate conditions that can be considered representative for the multi-year cultivation of durum wheat in the north-eastern Poland. The experiment was established on soil formed from sandy loam, in an area with a mildly undulated landscape and a slope of less than 2%. Durum wheat was grown in soil highly suitable for the production of rye (soil quality class IVa). The humus horizon had a slightly acidic pH in the range of 5.0 to 6.1. The phosphorus content of the arable layer was determined at 112–120 mg kg^−1^ of soil, potassium content was determined 145–182 mg kg^−1^ of soil, and magnesium content—at 52–53 mg kg^−1^ of soil. Winter oilseed rape was the preceding crop in the study years. Conventional tillage was applied. Two harrowing treatments were performed in the spring of each year. Before sowing, phosphorus (40% superphosphate) was applied at 35 kg ha^−1^, and potassium (60% potash salt) was applied at 83 kg ha^−1^. Nitrogen fertilizer (34% ammonium nitrate) was applied according to the methodology described in the experimental design.

The experiment had a strip-split plot design with three blocks. The experimental factors were: nitrogen fertilizer rate, sowing density and the application of the growth regulator. Nitrogen fertilizer was applied in the following rates and growth stages: no fertilization; 80 kg N ha^−1^ split into two applications: 50 kg N ha^−1^ before sowing and 30 kg N ha^−1^ in the 3rd node detectable stage (Zadoks growth stage Z33); 120 kg N ha^−1^ split into three applications: 50 kg N ha^−1^ before sowing, 30 kg N ha^−1^ in the 3rd node detectable stage (Z33), and 40 kg N ha^−1^ in the ear emergence stage (Z51). Durum wheat was sown at three densities of 350, 450 and 550 seeds m^−2^. The Medax Top 350 S.C. growth regulator (active ingredients: mepiquat chloride and prohexadione calcium) was applied at Z37-39. Treatments with various fertilization rates and sowing densities were randomly distributed in sub-blocks. Treatments without and with the growth regulator were randomly distributed in perpendicular strips. Plot area was 8.75 m^2^. Durum wheat was sown on 25 March in 2015 and on 29 March in 2016 and 2017.

In all treatments, weeds were controlled chemically with Chwastox Extra 300 SL (active ingredient: 2-methyl-4-chlorophenoxyacetic acid—MPCA potassium salt) in the tillering stage, and diseases were managed with Amistar 250 EC (active ingredient: azoxystrobin—a strobilurin compound) in the heading stage. Plants were protected against the cereal leaf beetle (*Oulema melanopa* L.) with Karate Zeon 050 CS (active ingredient: lambda-cyhalothrin—a pyrethroid compound) in the early heading stage.

The net photosynthetic rate (Pn), transpiration rate (E) and water use efficiency (WUE = Pn/E) were measured during each growing season with the LI-6400 Portable Photosynthesis System^66^ equipped with a 5 cm^2^ leaf chamber. Leaf temperature during the measurements ranged from 22 to 27 °C. The LAI was determined with the LI-COR LAI-2200 Plant Canopy Analyzer^67^. Chlorophyll content was measured in the center of the leaf blade with the SPAD-502 chlorophyll meter (Konica Minolta). SPAD values are dimensionless numerical values that are proportional to the total amount of chlorophyll in a leaf^26^. All parameters were measured in stages Z32 (2nd node detectable), Z45 (booting—flag leaf) and Z59 (end of heading: inflorescence fully emerged—flag leaf)^7^. All measurements were conducted between 9:00 a.m. and 12:00 p.m.

Immediately before harvest, plant lodging was evaluated on a 9-point scale, where 1 point denotes complete lodging, and 9 points denote the absence of lodging. Significant lodging was not observed; therefore, this parameter was not included in the model.

Twenty plants were collected from each plot for analyses of morphological traits (stem length and ear length) and yield components (number of kernels per ear and grain weight per ear). Thousand kernel weight was determined in samples of threshed grain from each plot. Durum wheat was harvested with a plot harvester in the fully ripe stage. Grain weight, straw weight and the harvest index ($HI=\frac{{Y}_{G}}{{Y}_{G}+{Y}_{S}}$, where ${Y}_{G}$ is grain weight and ${Y}_{S}$ is straw weight) were determined after harvest.

### Sources of variation

The effects of agricultural factors on physiological and biophysical parameters, yield components and yield were estimated by ANOVA which was performed separately for each of the three growth stages to determine differences between the experimental years, and for the three analyzed agronomic factors (nitrogen rate, sowing density and the application of the growth regulator) (Table 1). The fourth factor, i.e. phenological stages Z32, Z45 and Z59, was then incorporated into ANOVA. The treatment means were compared by Tukey’s HSD test at a significance level of *p* ≤ 0.05.

Eta-square η^2^ was estimated in ANOVA to measure the effect of different sources of variation, i.e. years, agricultural factors, phenological stages and random non-controlled factors, on the variables associated with plant physiology (Table 4).

**Table 4:** The notation for variables in Partial Least Square Path Modeling (PLS-PM).

Latent variable (symbol, name)	Manifest variable (symbol, name)		Unit
A Agronomic factors	GR	Application of the growth regulator	1 (yes), 0 (no)
	ND	Nitrogen doses	kg N ha^−1^
	SD	Sowing density	No of plants per m^2^
CC32 Climate conditions at Z32	Da32	Length of growth stage up to Z32	No of days
	Te32	Sum of temperatures up to Z32	°C
	Pr32	Sum of precipitation up to Z32	mm
PP32 Physiological processes at Z32	P32	Net photosynthetic rate at Z32	μmol(CO_2_) m^–2^ s^–1^
	E32	Transpiration rate at Z32	mmol(H_2_O)
BP32 Biophysical parameters at Z32	L32	Leaf area index at Z32	
	S32	Leaf greenness at Z32	SPAD
CC45 Climate conditions at Z45	Da45	Length of growth stage up to Z45	No of days
	Te45	Sum of temperatures up to Z45	°C
	Pr45	Sum of precipitation up to Z45	mm
PP45 Physiological processes at Z45	P45	Net photosynthetic rate at Z45	μmol(CO_2_) m^–2^ s^–1^
	E45	Transpiration rate at Z45	mmol(H_2_O)
BP45 Biophysical parameters at Z45	L32	Leaf area index at Z45	
	S45	Leaf greenness at Z45	SPAD
CC59 Climate conditions at Z59	Da59	Length of growth stage up to Z59	No of days
	Te59	Sum of temperatures up to Z59	°C
	Pr59	Sum of precipitation up to Z59	mm
PP59 Physiological processes at Z59	P59	Net photosynthetic rate at Z59	μmol(CO_2_) m^–2^ s^–1^
	E59	Transpiration rate at Z59	mmol(H_2_O)
BP59 Biophysical parameters at Z59	L59	Leaf area index at Z59	
	S59	Leaf greenness at Z59	SPAD
YC Yield components	SL	Stem length	cm
	EL	Ear length	cm
	KE	Number of kernels per ear	No
	KW	Grain weight per ear	g
CC Climate conditions during season	DAYS	Length of growth stage (entire season)	No of days
	TEMP	Sum of temperatures (entire season)	°C
	PREC	Sum of precipitation (entire season)	mm
BY Biological yield	GRAIN	Grain yield	t ha^−1^
	STRAW	Straw yield	t ha^−1^

### Path modeling

The relationship between biological yield (latent variable *Y*) versus agricultural factors, the physiological status of plants at critical stages of growth and development, and yield components (explanatory latent variables) was explored by partial least square path modelling (PLS-PM) (Table 4). The relationships between latent variables defined the inner model. Consequently, the latent variables were indirectly associated with the directly measured manifest variables defining the outer model to describe the relationships between latent and manifest variables.

**Figure 4:**
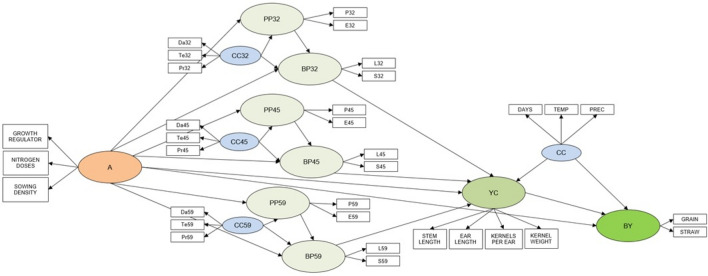
A generic diagram of the PLS-PM structural model describing the relationship between latent and manifest variables denoting *T. durum* plant growth and development (refer to Table 1 for the legend).

The applied PLS-PM structural model is presented in Fig. 4. It was assumed that agricultural practices contribute to optimal plant growth in critical phenological stages that affect the formation of yield components and, finally, biological yield. Hence, the model can support three successive working hypotheses:

1. An improvement in agricultural practices will foster the most favorable agricultural conditions in the critical stages of plant growth and development by affecting physiological processes and biophysical parameters of plants.
2. Favorable agricultural conditions for plant growth and development in critical phenological stages will promote the formation of yield components.
3. Improved formation of yield components will increase the biological yield.

The PLS-PM model features 13 latent variables (ellipses) that correspond to successive stages of biological yield formation, beginning from agricultural practices, through plant status in three phenological stages of growth and development, yield components, to biological yield. The model also contains 33 manifest variables (rectangles) that represent various aspects of latent variables. The measured parameters of plant growth and development were attributed to two latent variables. The first variable was referred to as physiological processes, and it was associated with the physiological processes of photosynthesis and transpiration. The second variable was referred to as biophysical parameters, and it was associated with physical measurements of leaf area and SPAD. The yield components latent variable consisted of direct measurements of stem length, ear length, number of kernels per ear, and grain weight per ear. The biological yield variable was associated with direct measurements of grain yield and straw yield. The model also implemented climate conditions during three stages of *T. durum* growth and development and in the entire season in each year of the experiment (length of a given stage in days, sum of temperatures, and sum of precipitation) that were associated with yield components and biological yield, respectively.

The developed model was used to estimate the following parameters: (1) outer weights (*w*_i_) and loadings (*l*_i_) of manifest variables denoting the relative importance of each originally measured manifest variable, i.e. the extent to which changes in manifest variables induced unitary changes in latent variables; (2) direct path coefficients *β*i between latent variables, and (3) contribution to variation in endogenous latent variables: physiological and biophysical parameters describing plant status in phenological stages, yield components and biological yield.

All statistical analyses were performed with the use of STATISTICA^68^ and XLSTAT^69^ packages.

## Supplementary Information

Supplementary Information.

## Data Availability

The data that support the findings of this study are available from the corresponding author J.G., upon reasonable request.
