# Supplementary material for: Modelling the growth, development and yield of Triticum durum Desf under the changes of climatic conditions in north-eastern Europe
Source: Sci Rep. 2021 Nov 5;11:21753. doi: 10.1038/s41598-021-01273-8 (PMC8571285; doi:10.1038/s41598-021-01273-8)
Supplement: Supplementary file 1 — Supplementary Information. [file 41598_2021_1273_MOESM1_ESM.pdf]

# Supplementary Information

# Modelling the growth, development and yield of *Triticum durum* Desf under the changes of climatic conditions in north-eastern Europe

Kamila S. Bożek[1], Krystyna Żuk-Gołaszewska[1], Anna Bochenek[2], Janusz Gołaszewski*[3] and Hazem M. Kalaji[4]

[1] Department of Agrotechnology and Agribusiness, Faculty of Agriculture and Forestry, University of Warmia and Mazury in Olsztyn, ul. Oczapowskiego 8, 10-719 Olsztyn, Poland

[2] Department of Plant Physiology, Genetics and Biotechnology, Faculty of Biology and Biotechnology, University of Warmia and Mazury in Olsztyn, ul. Oczapowskiego 1A, 10-719 Olsztyn, Poland

[3*] Department of Genetics, Plant Breeding and Bioresource Engineering, Faculty of Agriculture and Forestry, University of Warmia and Mazury in Olsztyn, Pl. Łódzki 3, 10-724 Olsztyn, Poland

[4] Department of Plant Physiology, Institute of Biology, Warsaw, University of Life Sciences SGGW, Warsaw, Poland

* Corresponding author: Janusz Gołaszewski (janusz.golaszewski@uwm.edu.pl).

# Table of content

Tables 1.1-1.6. The main and interaction effects of years, nitrogen rates and sowing density for SPAD and LAI values in the growth stages of T. durum (Zadoks 32, 45 and 59). .......... 3

Table 1.1 LAI(Z32) .......... 3

Table 1.2 SPAD(Z32) .......... 4

Table 1.3 LAI(Z45) .......... 5

Table 1.4 SPAD(Z45) .......... 6

Table 1.5 LAI(Z59) .......... 7

Table 1.6 SPAD(Z59) .......... 8

Tables 2.1-2.9. The main and interaction effects of year, the application of the growth regulator, nitrogen dose and sowing density for the net photosynthetic rate (Pn), transpiration rate (E) and instantaneous water use efficiency (WUE) in the growth stages of *T. durum* (Zadoks 32, 45 and 59). . 9

Table 2.1 Pn(Z32) (μmol($CO_2$) $m^{-2}s^{-1}$) .......... 9

Table 2.2 E(Z32) (mmol($H_2O$) .......... 10

Table 2.3 WUE(Z32) .......... 11

Table 2.4 Pn(Z45) (μmol($CO_2$) $m^{-2}s^{-1}$) .......... 12

Table 2.5 E(Z45) (mmol($H_2O$) .......... 13

Table 2.6 WUE(Z45) .......... 14

Table 2.7 Pn(Z59) (μmol($CO_2$) $m^{-2}s^{-1}$) .......... 15

Table 2.8 E(Z59) (mmol($H_2O$) .......... 16

Table 2.9 WUE(Z59) .......... 17

Tables 3.1-3.8. The main and interaction effects of years and agronomic factors for the yield components and yield of *T. durum*. .......... 18

Table 3.1 Stem length (cm) .......... 18

Table 3.2 Ear length (cm) .......... 19

Table 3.3 Kernels per ear .......... 20

Table 3.4 Kernels weight (g) .......... 21

Table 3.6 Straw weight (t $ha^{-1}$) .......... 23

Table 3.7 Harvest index .......... 24

Table 3.8 Biological yield (t $ha^{-1}$) .......... 25

Table 4. Outer weights and loadings of manifest variables. .......... 26

**Tables 1.1-1.6. The main and interaction effects of years, nitrogen rates and sowing density for SPAD and LAI values in the growth stages of T. durum (Zadoks 32, 45 and 59).**

| Symbols: | |
|---|---|
| LAI | Leaf area index |
| SPAD | SPAD values of chlorophyll meter |
| Z32, Z45, Z59 | Zadoks growth stages (Z32, Z45, Z59) |
| Y | Year |
| GR | Growth regulator (0 - no, 1 - yes) |
| ND | Nitrogen dose in kg $ha^{-1}$ |
| SD | Sowing density (No of plants/$m^2$) |
| HSD | Honest Significant Difference acc. to Tukey's T-test |

**Table 1.1 LAI(Z32)**

| ND | SD | 2015 | | 2016 | | 2017 | | 2015 | 2016 | 2017 | |
|---|---|---|---|---|---|---|---|---|---|---|---|
| | | R0 | R1 | R0 | R1 | R0 | R1 | | | | |
| | | YxGRxNDxSD | | | | | | YxNDxSD | | | NxSD |
| 0 | 350 | 0.97 | 1.17 | 0.87 | 0.76 | 0.74 | 0.45 | 1.07 | 0.82 | 0.59 | 0.83 |
| | 450 | 1.25 | 1.33 | 0.77 | 0.74 | 0.71 | 0.72 | 1.29 | 0.75 | 0.71 | 0.92 |
| | 550 | 1.00 | 1.05 | 1.07 | 0.73 | 0.81 | 0.73 | 1.02 | 0.90 | 0.77 | 0.90 |
| 80 | 350 | 1.32 | 1.49 | 0.93 | 1.03 | 1.69 | 2.07 | 1.40 | 0.98 | 1.88 | 1.42 |
| | 450 | 1.46 | 1.18 | 1.18 | 1.37 | 2.00 | 1.87 | 1.32 | 1.27 | 1.94 | 1.51 |
| | 550 | 1.45 | 1.26 | 1.52 | 1.34 | 1.72 | 2.03 | 1.35 | 1.43 | 1.87 | 1.55 |
| 120 | 350 | 1.49 | 1.35 | 1.09 | 1.54 | 1.62 | 1.22 | 1.42 | 1.32 | 1.42 | 1.39 |
| | 450 | 1.68 | 1.67 | 1.31 | 1.13 | 1.96 | 1.85 | 1.67 | 1.22 | 1.91 | 1.60 |
| | 550 | 1.43 | 1.86 | 1.39 | 1.42 | 1.73 | 1.77 | 1.65 | 1.41 | 1.75 | 1.60 |
| | | YxGRxND | | | | | | YxND (4.56) | | | ND |
| 0 | | 1.07 | 1.18 | 0.90 | 0.74 | 0.75 | 0.63 | 1.13 | 0.82 | 0.69 | 0.88 |
| 80 | | 1.41 | 1.31 | 1.21 | 1.25 | 1.80 | 1.99 | 1.36 | 1.23 | 1.90 | 1.49 |
| 120 | | 1.53 | 1.63 | 1.26 | 1.36 | 1.77 | 1.61 | 1.58 | 1.31 | 1.69 | 1.53 |
| | | YxGRxSD | | | | | | YxSD | | | SD |
| | 350 | 1.26 | 1.34 | 0.96 | 1.11 | 1.35 | 1.25 | 1.30 | 1.04 | 1.30 | 1.21 |
| | 450 | 1.46 | 1.39 | 1.08 | 1.08 | 1.56 | 1.48 | 1.43 | 1.08 | 1.52 | 1.34 |
| | 550 | 1.29 | 1.39 | 1.33 | 1.16 | 1.42 | 1.51 | 1.34 | 1.25 | 1.46 | 1.35 |
| | | YxGR | | | | | | Y | | | Grand mean |
| | | 1.34 | 1.37 | 1.12 | 1.12 | 1.44 | 1.41 | 1.36 | 1.12 | 1.43 | 1.30 |

**LAI(Z32)**

| Significant effect | HSD(alfa=0.05) |
|---|---|
| ND | 0.216 |
| Y*ND | 0.521 |
| SD | 0.056 |

**Table 1.2 SPAD(Z32)**

| ND | SD | 2015 | | 2016 | | 2017 | | 2015 | 2016 | 2017 | |
|---|---|---|---|---|---|---|---|---|---|---|---|
| | | R0 | R1 | R0 | R1 | R0 | R1 | | | | |
| | | YxGRxNDxSD | | | | | | YxNDxSD | | | NxSD |
| 0 | 350 | 45.88 | 44.98 | 44.31 | 43.59 | 47.62 | 47.32 | 45.43 | 43.95 | 47.47 | 45.62 |
| | 450 | 45.23 | 46.54 | 43.77 | 44.07 | 47.65 | 47.39 | 45.89 | 43.92 | 47.52 | 45.77 |
| | 550 | 46.91 | 46.39 | 43.26 | 43.02 | 47.31 | 47.35 | 46.65 | 43.14 | 47.33 | 45.71 |
| 80 | 350 | 49.86 | 51.24 | 53.00 | 47.77 | 48.26 | 47.81 | 50.55 | 50.38 | 48.04 | 49.66 |
| | 450 | 49.07 | 49.92 | 45.48 | 48.06 | 47.37 | 47.81 | 49.49 | 46.77 | 47.59 | 47.95 |
| | 550 | 46.82 | 46.67 | 50.12 | 49.53 | 47.65 | 47.79 | 46.74 | 49.83 | 47.72 | 48.10 |
| 120 | 350 | 49.14 | 48.66 | 51.47 | 52.10 | 48.43 | 48.00 | 48.90 | 51.78 | 48.21 | 49.63 |
| | 450 | 46.79 | 45.77 | 50.64 | 48.81 | 47.64 | 48.45 | 46.28 | 49.73 | 48.04 | 48.02 |
| | 550 | 50.13 | 47.22 | 50.79 | 49.52 | 48.20 | 48.17 | 48.68 | 50.16 | 48.18 | 49.01 |
| | | YxGRxND | | | | | | YxND (4.56) | | | ND |
| 0 | | 46.01 | 45.97 | 43.78 | 43.56 | 47.53 | 47.35 | 45.99 | 43.67 | 47.44 | 45.70 |
| 80 | | 48.58 | 49.28 | 49.53 | 48.45 | 47.76 | 47.81 | 48.93 | 48.99 | 47.78 | 48.57 |
| 120 | | 48.69 | 47.21 | 50.97 | 50.14 | 48.09 | 48.21 | 47.95 | 50.56 | 48.15 | 48.88 |
| | | YxGRxSD | | | | | | YxSD | | | SD |
| | 350 | 48.29 | 48.29 | 49.59 | 47.82 | 48.10 | 47.71 | 48.29 | 48.71 | 47.91 | 48.30 |
| | 450 | 47.03 | 47.41 | 46.63 | 46.98 | 47.55 | 47.88 | 47.22 | 46.80 | 47.72 | 47.25 |
| | 550 | 47.96 | 46.76 | 48.06 | 47.36 | 47.72 | 47.77 | 47.36 | 47.71 | 47.74 | 47.60 |
| | | YxGR | | | | | | Y | | | Grand mean |
| | | 47.76 | 47.49 | 48.09 | 47.39 | 47.79 | 47.79 | 47.62 | 47.74 | 47.79 | 47.72 |

**SPAD(Z32)**

| Significant effect | HSD(alfa=0.05) |
|---|---|
| ND | 1.373 |
| Y*ND | 3.312 |
| SD | 1.011 |

**Table 1.3 LAI(Z45)**

| ND | SD | 2015 | | 2016 | | 2017 | | 2015 | 2016 | 2017 | |
|---|---|---|---|---|---|---|---|---|---|---|---|
| | | R0 | R1 | R0 | R1 | R0 | R1 | | | | |
| | | YxGRxNDxSD | | | | | | YxNDxSD | | | NxSD |
| 0 | 350 | 1.54 | 1.33 | 1.37 | 1.35 | 1.48 | 1.33 | 1.44 | 1.36 | 1.41 | 1.40 |
| | 450 | 1.11 | 1.13 | 1.24 | 1.73 | 1.47 | 1.48 | 1.12 | 1.49 | 1.47 | 1.36 |
| | 550 | 1.86 | 1.11 | 1.78 | 1.47 | 1.52 | 1.48 | 1.49 | 1.63 | 1.50 | 1.54 |
| 80 | 350 | 1.71 | 1.17 | 1.83 | 2.11 | 2.01 | 2.21 | 1.44 | 1.97 | 2.11 | 1.84 |
| | 450 | 1.81 | 1.64 | 2.41 | 2.21 | 2.17 | 2.11 | 1.72 | 2.31 | 2.14 | 2.06 |
| | 550 | 1.20 | 1.25 | 1.70 | 2.35 | 2.02 | 2.19 | 1.22 | 2.03 | 2.11 | 1.78 |
| 120 | 350 | 1.16 | 1.39 | 2.32 | 2.31 | 1.97 | 1.75 | 1.28 | 2.31 | 1.86 | 1.81 |
| | 450 | 1.95 | 1.82 | 2.34 | 2.28 | 2.15 | 2.09 | 1.88 | 2.31 | 2.12 | 2.11 |
| | 550 | 1.54 | 1.51 | 2.05 | 2.11 | 2.03 | 2.05 | 1.52 | 2.08 | 2.04 | 1.88 |
| | | YxGRxND | | | | | | YxND (4.56) | | | ND |
| 0 | | 1.50 | 1.19 | 1.46 | 1.52 | 1.49 | 1.43 | 1.35 | 1.49 | 1.46 | 1.43 |
| 80 | | 1.57 | 1.35 | 1.98 | 2.22 | 2.07 | 2.17 | 1.46 | 2.10 | 2.12 | 1.89 |
| 120 | | 1.55 | 1.57 | 2.24 | 2.23 | 2.05 | 1.96 | 1.56 | 2.23 | 2.01 | 1.93 |
| | | YxGRxSD | | | | | | YxSD | | | SD |
| | 350 | 1.47 | 1.30 | 1.84 | 1.92 | 1.82 | 1.76 | 1.38 | 1.88 | 1.79 | 1.69 |
| | 450 | 1.62 | 1.53 | 2.00 | 2.07 | 1.93 | 1.89 | 1.58 | 2.04 | 1.91 | 1.84 |
| | 550 | 1.53 | 1.29 | 1.84 | 1.98 | 1.86 | 1.91 | 1.41 | 1.91 | 1.88 | 1.73 |
| | | YxGR | | | | | | Y | | | Grand mean |
| | | 1.54 | 1.37 | 1.89 | 1.99 | 1.87 | 1.85 | 1.46 | 1.94 | 1.86 | 1.75 |

**LAI(Z45)**

| Significant effect | HSD(alfa=0.05) |
|---|---|
| Y | 0.344 |
| ND | 0.254 |

**Table 1.4 SPAD(Z45)**

| ND | SD | 2015 | | 2016 | | 2017 | | 2015 | 2016 | 2017 | |
|---|---|---|---|---|---|---|---|---|---|---|---|
| | | R0 | R1 | R0 | R1 | R0 | R1 | | | | |
| | | YxGRxNDxSD | | | | | | YxNDxSD | | | NxSD |
| 0 | 350 | 50.48 | 43.90 | 37.60 | 43.49 | 41.06 | 39.37 | 47.19 | 40.54 | 40.21 | 42.65 |
| | 450 | 47.64 | 47.01 | 38.18 | 40.34 | 41.22 | 39.71 | 47.33 | 39.26 | 40.47 | 42.35 |
| | 550 | 48.67 | 46.21 | 39.67 | 37.72 | 39.30 | 39.50 | 47.44 | 38.69 | 39.40 | 41.84 |
| 80 | 350 | 50.19 | 53.69 | 41.18 | 44.88 | 44.70 | 42.16 | 51.94 | 43.03 | 43.43 | 46.13 |
| | 450 | 49.01 | 48.52 | 40.78 | 43.80 | 39.63 | 42.16 | 48.77 | 42.29 | 40.89 | 43.98 |
| | 550 | 48.77 | 51.38 | 42.82 | 43.69 | 41.20 | 42.01 | 50.07 | 43.26 | 41.61 | 44.98 |
| 120 | 350 | 49.02 | 50.93 | 43.36 | 41.53 | 45.64 | 43.23 | 49.98 | 42.44 | 44.44 | 45.62 |
| | 450 | 50.73 | 50.79 | 41.94 | 43.27 | 41.14 | 45.80 | 50.76 | 42.61 | 43.47 | 45.61 |
| | 550 | 47.28 | 49.93 | 44.23 | 42.62 | 44.34 | 44.18 | 48.61 | 43.43 | 44.26 | 45.43 |
| | | YxGRxND | | | | | | YxND (4.56) | | | ND |
| 0 | | 48.93 | 45.71 | 38.48 | 40.52 | 40.53 | 39.53 | 47.32 | 39.50 | 40.03 | 42.28 |
| 80 | | 49.32 | 51.20 | 41.59 | 44.12 | 41.84 | 42.11 | 50.26 | 42.86 | 41.98 | 45.03 |
| 120 | | 49.01 | 50.55 | 43.18 | 42.47 | 43.71 | 44.40 | 49.78 | 42.83 | 44.06 | 45.55 |
| | | YxGRxSD | | | | | | YxSD | | | SD |
| | 350 | 49.90 | 49.51 | 40.71 | 43.30 | 43.80 | 41.59 | 49.70 | 42.01 | 42.69 | 44.80 |
| | 450 | 49.13 | 48.77 | 40.30 | 42.47 | 40.67 | 42.56 | 48.95 | 41.39 | 41.61 | 43.98 |
| | 550 | 48.24 | 49.17 | 42.24 | 41.34 | 41.61 | 41.90 | 48.71 | 41.79 | 41.76 | 44.08 |
| | | YxGR | | | | | | Y | | | Grand mean |
| | | 49.09 | 49.15 | 41.08 | 42.37 | 42.03 | 42.01 | 49.12 | 41.73 | 42.02 | 44.29 |

**SPAD(Z45)**

| Significant effect | HSD(alfa=0.05) |
|---|---|
| Y | 1.735 |
| ND | 1.839 |

**Table 1.5 LAI(Z59)**

| ND | SD | 2015 | | 2016 | | 2017 | | 2015 | 2016 | 2017 | |
|---|---|---|---|---|---|---|---|---|---|---|---|
| | | R0 | R1 | R0 | R1 | R0 | R1 | | | | |
| | | YxGRxNDxSD | | | | | | YxNDxSD | | | NxSD |
| 0 | 350 | 1.49 | 1.81 | 1.41 | 1.31 | 1.40 | 1.33 | 1.65 | 1.36 | 1.37 | 1.46 |
| | 450 | 1.52 | 1.62 | 1.28 | 1.55 | 1.35 | 1.17 | 1.57 | 1.42 | 1.26 | 1.42 |
| | 550 | 1.72 | 1.67 | 1.96 | 1.70 | 1.44 | 1.47 | 1.69 | 1.83 | 1.46 | 1.66 |
| 80 | 350 | 1.89 | 2.17 | 2.11 | 2.13 | 2.06 | 2.08 | 2.03 | 2.12 | 2.07 | 2.07 |
| | 450 | 1.83 | 1.99 | 2.69 | 2.41 | 2.29 | 2.83 | 1.91 | 2.55 | 2.56 | 2.34 |
| | 550 | 1.99 | 1.74 | 2.61 | 2.53 | 2.41 | 2.57 | 1.87 | 2.57 | 2.49 | 2.31 |
| 120 | 350 | 1.65 | 1.81 | 2.47 | 2.21 | 2.36 | 2.39 | 1.73 | 2.34 | 2.38 | 2.15 |
| | 450 | 1.59 | 1.77 | 2.79 | 2.10 | 2.67 | 2.25 | 1.68 | 2.44 | 2.46 | 2.20 |
| | 550 | 1.74 | 2.20 | 2.52 | 2.71 | 2.20 | 2.22 | 1.97 | 2.62 | 2.21 | 2.27 |
| | | YxGRxND | | | | | | YxND (4.56) | | | ND |
| 0 | | 1.57 | 1.70 | 1.55 | 1.52 | 1.40 | 1.32 | 1.64 | 1.54 | 1.36 | 1.51 |
| 80 | | 1.90 | 1.97 | 2.47 | 2.36 | 2.25 | 2.49 | 1.93 | 2.42 | 2.37 | 2.24 |
| 120 | | 1.66 | 1.93 | 2.59 | 2.34 | 2.41 | 2.29 | 1.79 | 2.47 | 2.35 | 2.20 |
| | | YxGRxSD | | | | | | YxSD | | | SD |
| | 350 | 1.67 | 1.93 | 2.00 | 1.88 | 1.94 | 1.93 | 1.80 | 1.94 | 1.94 | 1.89 |
| | 450 | 1.65 | 1.79 | 2.25 | 2.02 | 2.10 | 2.09 | 1.72 | 2.14 | 2.10 | 1.98 |
| | 550 | 1.81 | 1.87 | 2.37 | 2.31 | 2.02 | 2.08 | 1.84 | 2.34 | 2.05 | 2.08 |
| | | YxGR | | | | | | Y | | | Grand mean |
| | | 1.71 | 1.87 | 2.21 | 2.07 | 2.02 | 2.03 | 1.79 | 2.14 | 2.03 | 1.99 |

**LAI(Z59)**

| Significant effect | HSD(alfa=0.05) |
|---|---|
| Y | 1.008 |
| ND | 0.168 |
| Y*ND | 0.405 |
| SD | 0.150 |

**Table 1.6 SPAD(Z59)**

| ND | SD | 2015 | | 2016 | | 2017 | | 2015 | 2016 | 2017 | |
|---|---|---|---|---|---|---|---|---|---|---|---|
| | | R0 | R1 | R0 | R1 | R0 | R1 | | | | |
| | | YxGRxNDxSD | | | | | | YxNDxSD | | | NxSD |
| 0 | 350 | 56.30 | 55.97 | 44.72 | 43.80 | 40.00 | 45.33 | 56.13 | 44.26 | 42.67 | 47.69 |
| | 450 | 54.64 | 54.21 | 42.84 | 47.23 | 36.82 | 40.11 | 54.43 | 45.04 | 38.47 | 45.98 |
| | 550 | 54.26 | 53.17 | 44.78 | 44.81 | 36.82 | 34.36 | 53.71 | 44.79 | 35.59 | 44.70 |
| 80 | 350 | 55.57 | 56.08 | 50.40 | 48.13 | 48.09 | 49.13 | 55.82 | 49.27 | 48.61 | 51.23 |
| | 450 | 54.27 | 57.44 | 49.16 | 47.43 | 46.20 | 43.36 | 55.86 | 48.29 | 44.78 | 49.64 |
| | 550 | 55.41 | 53.58 | 48.43 | 46.49 | 47.21 | 46.00 | 54.49 | 47.46 | 46.61 | 49.52 |
| 120 | 350 | 57.34 | 53.94 | 53.16 | 52.51 | 47.89 | 52.58 | 55.64 | 52.83 | 50.23 | 52.90 |
| | 450 | 54.28 | 57.22 | 52.14 | 46.79 | 47.70 | 48.02 | 55.75 | 49.47 | 47.86 | 51.03 |
| | 550 | 54.69 | 54.07 | 53.32 | 49.36 | 45.49 | 48.56 | 54.38 | 51.34 | 47.02 | 50.91 |
| | | YxGRxND | | | | | | YxND (4.56) | | | ND |
| 0 | | 55.07 | 54.45 | 44.11 | 45.28 | 37.88 | 39.93 | 54.76 | 44.70 | 38.91 | 46.12 |
| 80 | | 55.08 | 55.70 | 49.33 | 47.35 | 47.17 | 46.16 | 55.39 | 48.34 | 46.66 | 50.13 |
| 120 | | 55.44 | 55.08 | 52.87 | 49.55 | 47.03 | 49.72 | 55.26 | 51.21 | 48.37 | 51.61 |
| | | YxGRxSD | | | | | | YxSD | | | SD |
| | 350 | 56.40 | 55.33 | 49.43 | 48.15 | 45.33 | 49.01 | 55.87 | 48.79 | 47.17 | 50.61 |
| | 450 | 54.40 | 56.29 | 48.05 | 47.15 | 43.57 | 43.83 | 55.34 | 47.60 | 43.70 | 48.88 |
| | 550 | 54.79 | 53.60 | 48.84 | 46.89 | 43.17 | 42.97 | 54.19 | 47.86 | 43.07 | 48.38 |
| | | YxGR | | | | | | Y | | | Grand mean |
| | | 55.20 | 55.08 | 48.77 | 47.40 | 44.02 | 45.27 | 55.14 | 48.08 | 44.65 | 49.29 |

**SPAD(Z59)**

| Significant effect | HSD(alfa=0.05) | |
|---|---|---|
| Y | 1.008 | 2.618 |
| ND | 0.168 | 1.974 |
| Y*ND | 0.405 | 4.759 |
| SD | 0.150 | 1.464 |

**Tables 2.1-2.9. The main and interaction effects of year, the application of the growth regulator, nitrogen dose and sowing density for the net photosynthetic rate (Pn), transpiration rate (E) and instantaneous water use efficiency (WUE) in the growth stages of *T. durum* (Zadoks 32, 45 and 59).**

Symbols:

| | |
|---|---|
| Pn | Net photosynthesis |
| E | Evapotranspiration |
| WUE | Water use efficiency |
| Y | Year |
| GR | Growth regulator (0 - no, 1 - yes) |
| ND | Nitrogen dose in kg $ha^{-1}$ |
| SD | Sowing density (No of plants/$m^2$) |
| GS | Zadoks growth stages (Z32, Z45, Z59) |
| HSD | Honest Significant Difference acc. to Tukey's T-test |

**Table 2.1 Pn(Z32) (µmol($CO_2$) $m^{-2}s^{-1}$)**

| ND | SD | 2015 | | 2016 | | 2017 | | 2015 | 2016 | 2017 | |
|---|---|---|---|---|---|---|---|---|---|---|---|
| | | R0 | R1 | R0 | R1 | R0 | R1 | | | | |
| | | YxGRxNDxSD | | | | | | YxNDxSD | | | NxSD |
| 0 | 350 | 33.5 | 40.9 | 25.4 | 24.0 | 28.6 | 31.7 | 37.2 | 24.7 | 30.2 | 30.7 |
| | 450 | 38.0 | 40.9 | 25.8 | 23.4 | 27.4 | 27.6 | 39.5 | 24.6 | 27.5 | 30.5 |
| | 550 | 28.8 | 37.1 | 24.7 | 24.9 | 27.8 | 24.2 | 33.0 | 24.8 | 26.0 | 27.9 |
| 80 | 350 | 32.9 | 30.0 | 27.4 | 27.3 | 24.9 | 30.4 | 31.4 | 27.4 | 27.7 | 28.8 |
| | 450 | 32.5 | 34.4 | 30.2 | 25.1 | 26.3 | 28.2 | 33.5 | 27.6 | 27.3 | 29.5 |
| | 550 | 37.9 | 32.5 | 27.2 | 29.2 | 29.5 | 30.1 | 35.2 | 28.2 | 29.8 | 31.1 |
| 120 | 350 | 36.7 | 40.0 | 28.4 | 19.1 | 29.5 | 31.4 | 38.4 | 23.8 | 30.4 | 30.9 |
| | 450 | 33.0 | 35.7 | 25.4 | 28.6 | 28.1 | 27.1 | 34.4 | 27.0 | 27.6 | 29.6 |
| | 550 | 28.2 | 32.4 | 27.9 | 27.4 | 22.6 | 32.3 | 30.3 | 27.6 | 27.4 | 28.5 |
| | | YxGRxND | | | | | | YxND (4.56) | | | ND |
| 0 | | 33.4 | 39.6 | 25.3 | 24.1 | 27.9 | 27.8 | 36.5 | 24.7 | 27.9 | 29.7 |
| 80 | | 34.4 | 32.3 | 28.3 | 27.2 | 26.9 | 29.6 | 33.4 | 27.7 | 28.2 | 29.8 |
| 120 | | 32.6 | 36.1 | 27.2 | 25.0 | 26.7 | 30.2 | 34.3 | 26.1 | 28.5 | 29.7 |
| | | YxGRxSD | | | | | | YxSD | | | SD |
| | 350 | 34.3 | 37.0 | 27.1 | 23.5 | 27.6 | 31.2 | 35.7 | 25.3 | 29.4 | 30.1 |
| | 450 | 34.5 | 37.0 | 27.1 | 25.7 | 27.2 | 27.6 | 35.8 | 26.4 | 27.4 | 29.9 |
| | 550 | 31.6 | 34.0 | 26.6 | 27.2 | 26.6 | 28.9 | 32.8 | 26.9 | 27.8 | 29.2 |
| | | YxGR | | | | | | Y | | | Grand mean |
| | | 33.5 | 36.0 | 26.9 | 25.4 | 27.2 | 29.2 | 34.7 | 26.2 | 28.2 | 29.7 |

**Pn(Z32)**

| Significant effect | HSD(alfa=0.05) |
|---|---|
| Y | 7.641 |

**Table 2.2 E(Z32) (mmol($H_2O$)**

| ND | SD | 2015 | | 2016 | | 2017 | | 2015 | 2016 | 2017 | |
|---|---|---|---|---|---|---|---|---|---|---|---|
| | | R0 | R1 | R0 | R1 | R0 | R1 | | | | |
| | | YxGRxNDxSD | | | | | | YxNDxSD | | | NxSD |
| 0 | 350 | 2.17 | 1.80 | 2.00 | 0.60 | 2.20 | 1.57 | 1.98 | 1.30 | 1.88 | 1.72 |
| | 450 | 2.50 | 3.13 | 3.00 | 0.63 | 2.20 | 1.47 | 2.82 | 1.82 | 1.83 | 2.16 |
| | 550 | 1.83 | 2.67 | 1.60 | 0.57 | 1.87 | 1.63 | 2.25 | 1.08 | 1.75 | 1.69 |
| 80 | 350 | 1.03 | 1.27 | 1.13 | 1.40 | 2.30 | 1.63 | 1.15 | 1.27 | 1.97 | 1.46 |
| | 450 | 1.67 | 1.73 | 0.97 | 1.97 | 1.97 | 1.57 | 1.70 | 1.47 | 1.77 | 1.64 |
| | 550 | 1.60 | 1.37 | 1.50 | 1.80 | 1.97 | 1.50 | 1.48 | 1.65 | 1.73 | 1.62 |
| 120 | 350 | 1.93 | 1.87 | 1.90 | 1.20 | 1.40 | 1.30 | 1.90 | 1.55 | 1.35 | 1.60 |
| | 450 | 1.87 | 1.27 | 0.93 | 0.77 | 1.10 | 1.07 | 1.57 | 0.85 | 1.08 | 1.17 |
| | 550 | 1.10 | 1.10 | 1.83 | 0.97 | 1.47 | 1.23 | 1.10 | 1.40 | 1.35 | 1.28 |
| | | YxGRxND | | | | | | YxND (4.56) | | | ND |
| 0 | | 2.17 | 2.53 | 2.20 | 0.60 | 2.09 | 1.56 | 2.35 | 1.40 | 1.82 | 1.86 |
| 80 | | 1.43 | 1.46 | 1.20 | 1.72 | 2.08 | 1.57 | 1.44 | 1.46 | 1.82 | 1.58 |
| 120 | | 1.63 | 1.41 | 1.56 | 0.98 | 1.32 | 1.20 | 1.52 | 1.27 | 1.26 | 1.35 |
| | | YxGRxSD | | | | | | YxSD | | | SD |
| | 350 | 1.71 | 1.64 | 1.68 | 1.07 | 1.97 | 1.50 | 1.68 | 1.37 | 1.73 | 1.59 |
| | 450 | 2.01 | 2.04 | 1.63 | 1.12 | 1.76 | 1.37 | 2.03 | 1.38 | 1.56 | 1.66 |
| | 550 | 1.51 | 1.71 | 1.64 | 1.11 | 1.77 | 1.46 | 1.61 | 1.38 | 1.61 | 1.53 |
| | | YxGR | | | | | | Y | | | Grand mean |
| | | 1.74 | 1.80 | 1.65 | 1.10 | 1.83 | 1.44 | 1.77 | 1.38 | 1.64 | 1.59 |

**E(Z32)**

| Significant effect | HSD(alfa=0.05) |
|---|---|
| Y*GR*ND | 1.637 |
| ND*SD | 0.665 |

**Table 2.3 WUE(Z32)**

| ND | SD | 2015 | | 2016 | | 2017 | | 2015 | 2016 | 2017 | |
|---|---|---|---|---|---|---|---|---|---|---|---|
| | | R0 | R1 | R0 | R1 | R0 | R1 | | | | |
| | | YxGRxNDxSD | | | | | | YxNDxSD | | | NxSD |
| 0 | 350 | 18.1 | 22.7 | 13.2 | 40.1 | 15.6 | 25.6 | 20.4 | 26.6 | 20.6 | 22.5 |
| | 450 | 16.1 | 13.4 | 9.0 | 37.7 | 24.9 | 25.3 | 14.8 | 23.4 | 25.1 | 21.1 |
| | 550 | 21.7 | 17.3 | 17.7 | 44.2 | 19.0 | 20.8 | 19.5 | 30.9 | 19.9 | 23.4 |
| 80 | 350 | 33.0 | 27.5 | 28.3 | 19.9 | 12.0 | 25.9 | 30.2 | 24.1 | 18.9 | 24.4 |
| | 450 | 20.9 | 20.6 | 42.1 | 14.6 | 14.1 | 28.9 | 20.8 | 28.4 | 21.5 | 23.5 |
| | 550 | 24.8 | 26.2 | 26.7 | 18.7 | 16.2 | 24.2 | 25.5 | 22.7 | 20.2 | 22.8 |
| 120 | 350 | 20.2 | 22.6 | 32.1 | 18.6 | 25.5 | 24.7 | 21.4 | 25.4 | 25.1 | 24.0 |
| | 450 | 17.5 | 37.0 | 27.5 | 39.0 | 26.7 | 25.8 | 27.2 | 33.3 | 26.2 | 28.9 |
| | 550 | 29.5 | 38.9 | 24.4 | 29.9 | 19.6 | 29.9 | 34.2 | 27.2 | 24.7 | 28.7 |
| | | YxGRxND | | | | | | YxND (4.56) | | | ND |
| 0 | | 18.6 | 17.8 | 13.3 | 40.7 | 19.8 | 23.9 | 18.2 | 27.0 | 21.9 | 22.4 |
| 80 | | 26.2 | 24.8 | 32.4 | 17.7 | 14.1 | 26.3 | 25.5 | 25.1 | 20.2 | 23.6 |
| 120 | | 22.4 | 32.8 | 28.0 | 29.2 | 23.9 | 26.8 | 27.6 | 28.6 | 25.4 | 27.2 |
| | | YxGRxSD | | | | | | YxSD | | | SD |
| | 350 | 23.8 | 24.2 | 24.5 | 26.2 | 17.7 | 25.4 | 24.0 | 25.4 | 21.5 | 23.6 |
| | 450 | 18.2 | 23.7 | 26.2 | 30.5 | 21.9 | 26.7 | 20.9 | 28.3 | 24.3 | 24.5 |
| | 550 | 25.3 | 27.4 | 22.9 | 30.9 | 18.2 | 25.0 | 26.4 | 26.9 | 21.6 | 25.0 |
| | | YxGR | | | | | | Y | | | Grand mean |
| | | 22.4 | 25.1 | 24.6 | 29.2 | 19.3 | 25.7 | 23.8 | 26.9 | 22.5 | 24.4 |

**WUE(Z32)**

| Significant effect | HSD(alfa=0.05) |
|---|---|
| Y*GR*ND | 27.630 |

**Table 2.4 Pn(Z45) (μmol($CO_2$) $m^{-2}s^{-1}$)**

| ND | SD | 2015 | | 2016 | | 2017 | | 2015 | 2016 | 2017 | |
|---|---|---|---|---|---|---|---|---|---|---|---|
| | | R0 | R1 | R0 | R1 | R0 | R1 | | | | |
| | | YxGRxNDxSD | | | | | | YxNDxSD | | | NxSD |
| 0 | 350 | 37.9 | 34.1 | 29.7 | 31.9 | 26.5 | 23.7 | 36.0 | 30.8 | 25.1 | 30.6 |
| | 450 | 33.1 | 32.8 | 33.4 | 26.3 | 28.2 | 26.2 | 32.9 | 29.9 | 27.2 | 30.0 |
| | 550 | 32.8 | 33.2 | 29.5 | 28.8 | 25.9 | 22.7 | 33.0 | 29.2 | 24.3 | 28.8 |
| 80 | 350 | 32.5 | 34.1 | 33.4 | 33.9 | 20.5 | 22.3 | 33.3 | 33.7 | 21.4 | 29.5 |
| | 450 | 36.1 | 30.3 | 35.7 | 34.0 | 23.0 | 26.9 | 33.2 | 34.8 | 24.9 | 31.0 |
| | 550 | 36.3 | 29.1 | 29.5 | 33.5 | 23.8 | 27.3 | 32.7 | 31.5 | 25.6 | 29.9 |
| 120 | 350 | 29.7 | 31.5 | 33.1 | 26.5 | 26.8 | 25.1 | 30.6 | 29.8 | 26.0 | 28.8 |
| | 450 | 32.6 | 36.2 | 28.0 | 23.8 | 26.5 | 28.5 | 34.4 | 25.9 | 27.5 | 29.3 |
| | 550 | 33.8 | 35.5 | 27.0 | 23.4 | 26.9 | 29.2 | 34.7 | 25.2 | 28.1 | 29.3 |
| | | YxGRxND | | | | | | YxND (4.56) | | | ND |
| 0 | | 34.6 | 33.3 | 30.9 | 29.0 | 26.9 | 24.2 | 34.0 | 30.0 | 25.5 | 29.8 |
| 80 | | 35.0 | 31.2 | 32.8 | 33.8 | 22.5 | 25.5 | 33.1 | 33.3 | 24.0 | 30.1 |
| 120 | | 32.0 | 34.4 | 29.3 | 24.6 | 26.7 | 27.6 | 33.2 | 26.9 | 27.2 | 29.1 |
| | | YxGRxSD | | | | | | YxSD | | | SD |
| | 350 | 33.4 | 33.2 | 32.1 | 30.8 | 24.6 | 23.7 | 33.3 | 31.4 | 24.2 | 29.6 |
| | 450 | 33.9 | 33.1 | 32.4 | 28.0 | 25.9 | 27.2 | 33.5 | 30.2 | 26.5 | 30.1 |
| | 550 | 34.3 | 32.6 | 28.7 | 28.6 | 25.5 | 26.4 | 33.4 | 28.6 | 26.0 | 29.3 |
| | | YxGR | | | | | | Y | | | Grand mean |
| | | 33.9 | 33.0 | 31.0 | 29.1 | 25.4 | 25.8 | 33.4 | 30.1 | 28.2 | 29.7 |

**Pn(Z45)**

| Significant effect | HSD(alfa=0.05) |
|---|---|
| No significant effects | |

**Table 2.5 E(Z45) (mmol($H_2O$))**

| ND | SD | 2015 | | 2016 | | 2017 | | 2015 | 2016 | 2017 | |
|---|---|---|---|---|---|---|---|---|---|---|---|
| | | R0 | R1 | R0 | R1 | R0 | R1 | | | | |
| | | YxGRxNDxSD | | | | | | YxNDxSD | | | NxSD |
| 0 | 350 | 2.00 | 1.77 | 3.20 | 2.97 | 3.53 | 3.40 | 1.88 | 3.08 | 3.47 | 2.81 |
| | 450 | 2.43 | 2.10 | 3.00 | 2.00 | 2.83 | 3.10 | 2.27 | 2.50 | 2.97 | 2.58 |
| | 550 | 1.70 | 1.27 | 2.60 | 3.23 | 3.30 | 3.10 | 1.48 | 2.92 | 3.20 | 2.53 |
| 80 | 350 | 1.13 | 2.03 | 3.30 | 2.47 | 3.27 | 3.40 | 1.58 | 2.88 | 3.33 | 2.60 |
| | 450 | 0.97 | 2.23 | 2.60 | 2.63 | 3.93 | 3.80 | 1.60 | 2.62 | 3.87 | 2.69 |
| | 550 | 1.00 | 2.10 | 2.50 | 3.57 | 3.80 | 3.43 | 1.55 | 3.03 | 3.62 | 2.73 |
| 120 | 350 | 1.50 | 1.33 | 2.10 | 2.10 | 2.47 | 2.57 | 1.42 | 2.10 | 2.52 | 2.01 |
| | 450 | 1.47 | 1.63 | 1.47 | 2.83 | 3.00 | 3.43 | 1.55 | 2.15 | 3.22 | 2.31 |
| | 550 | 1.57 | 1.33 | 2.43 | 2.57 | 2.87 | 3.47 | 1.45 | 2.50 | 3.17 | 2.37 |
| | | YxGRxND | | | | | | YxND (4.56) | | | ND |
| 0 | | 2.04 | 1.71 | 2.93 | 2.73 | 3.22 | 3.20 | 1.88 | 2.83 | 3.21 | 2.64 |
| 80 | | 1.03 | 2.12 | 2.80 | 2.89 | 3.67 | 3.54 | 1.58 | 2.84 | 3.61 | 2.68 |
| 120 | | 1.51 | 1.43 | 2.00 | 2.50 | 2.78 | 3.16 | 1.47 | 2.25 | 2.97 | 2.23 |
| | | YxGRxSD | | | | | | YxSD | | | SD |
| | 350 | 1.54 | 1.71 | 2.87 | 2.51 | 3.09 | 3.12 | 1.63 | 2.69 | 3.11 | 2.47 |
| | 450 | 1.62 | 1.99 | 2.36 | 2.49 | 3.26 | 3.44 | 1.81 | 2.42 | 3.35 | 2.53 |
| | 550 | 1.42 | 1.57 | 2.51 | 3.12 | 3.32 | 3.33 | 1.49 | 2.82 | 3.33 | 2.55 |
| | | YxGR | | | | | | Y | | | Grand mean |
| | | 1.53 | 1.76 | 2.58 | 2.71 | 3.22 | 3.30 | 1.64 | 2.64 | 3.26 | 2.52 |

**E(Z45)**

| Significant effect | HSD(alfa=0.05) |
|---|---|
| Y | 0.760 |
| ND | 0.433 |

**Table 2.6 WUE(Z45)**

| ND | SD | 2015 | | 2016 | | 2017 | | 2015 | 2016 | 2017 | |
|---|---|---|---|---|---|---|---|---|---|---|---|
| | | R0 | R1 | R0 | R1 | R0 | R1 | | | | |
| | | YxGRxNDxSD | | | | | | YxNDxSD | | | NxSD |
| 0 | 350 | 19.2 | 25.9 | 9.2 | 11.2 | 7.5 | 7.2 | 22.6 | 10.2 | 7.4 | 13.4 |
| | 450 | 15.2 | 16.8 | 11.3 | 13.6 | 10.4 | 8.5 | 16.0 | 12.5 | 9.5 | 12.6 |
| | 550 | 21.9 | 33.8 | 11.7 | 10.3 | 8.1 | 7.6 | 27.8 | 11.0 | 7.9 | 15.6 |
| 80 | 350 | 34.0 | 18.8 | 10.5 | 15.4 | 6.5 | 6.6 | 26.4 | 12.9 | 6.6 | 15.3 |
| | 450 | 40.1 | 14.4 | 14.6 | 12.9 | 6.2 | 7.0 | 27.2 | 13.8 | 6.6 | 15.9 |
| | 550 | 39.8 | 16.6 | 15.2 | 9.4 | 6.3 | 8.9 | 28.2 | 12.3 | 7.6 | 16.0 |
| 120 | 350 | 23.6 | 25.3 | 16.8 | 13.3 | 12.3 | 10.5 | 24.5 | 15.1 | 11.4 | 17.0 |
| | 450 | 27.2 | 22.8 | 20.6 | 8.5 | 9.3 | 8.2 | 25.0 | 14.5 | 8.7 | 16.1 |
| | 550 | 26.7 | 27.3 | 11.2 | 9.5 | 10.2 | 8.4 | 27.0 | 10.4 | 9.3 | 15.5 |
| | | YxGRxND | | | | | | YxND (4.56) | | | ND |
| 0 | | 18.8 | 25.5 | 10.7 | 11.7 | 8.7 | 7.8 | 22.1 | 11.2 | 8.2 | 13.9 |
| 80 | | 37.9 | 16.6 | 13.4 | 12.6 | 6.3 | 7.5 | 27.3 | 13.0 | 6.9 | 15.7 |
| 120 | | 25.8 | 25.1 | 16.2 | 10.4 | 10.6 | 9.0 | 25.5 | 13.3 | 9.8 | 16.2 |
| | | YxGRxSD | | | | | | YxSD | | | SD |
| | 350 | 25.6 | 23.3 | 12.2 | 13.3 | 8.8 | 8.1 | 24.5 | 12.7 | 8.4 | 15.2 |
| | 450 | 27.5 | 18.0 | 15.5 | 11.7 | 8.6 | 7.9 | 22.7 | 13.6 | 8.3 | 14.9 |
| | 550 | 29.4 | 25.9 | 12.7 | 9.7 | 8.2 | 8.3 | 27.7 | 11.2 | 8.2 | 15.7 |
| | | YxGR | | | | | | Y | | | Grand mean |
| | | 27.5 | 22.4 | 13.5 | 11.6 | 8.5 | 8.1 | 25.0 | 12.5 | 8.3 | 15.3 |

**WUE(Z45)**

| Significant effect | HSD(alfa=0.05) |
|---|---|
| Y | 9.806 |

**Table 2.7 Pn(Z59) (μmol($CO_2$) $m^{-2}s^{-1}$)**

| ND | SD | 2015 | | 2016 | | 2017 | | 2015 | 2016 | 2017 | |
|---|---|---|---|---|---|---|---|---|---|---|---|
| | | R0 | R1 | R0 | R1 | R0 | R1 | | | | |
| | | YxGRxNDxSD | | | | | | YxNDxSD | | | NxSD |
| 0 | 350 | 39.7 | 45.8 | 34.3 | 36.7 | 28.3 | 26.1 | 42.8 | 35.5 | 27.2 | 35.1 |
| | 450 | 41.7 | 46.5 | 30.6 | 35.7 | 23.9 | 24.7 | 44.1 | 33.1 | 24.3 | 33.8 |
| | 550 | 44.0 | 41.6 | 34.4 | 30.9 | 25.1 | 25.2 | 42.8 | 32.7 | 25.1 | 33.5 |
| 80 | 350 | 42.2 | 41.8 | 37.1 | 37.9 | 23.9 | 24.9 | 42.0 | 37.5 | 24.4 | 34.6 |
| | 450 | 44.0 | 43.8 | 36.0 | 33.7 | 27.3 | 24.6 | 43.9 | 34.9 | 25.9 | 34.9 |
| | 550 | 46.6 | 44.6 | 39.8 | 36.7 | 22.6 | 24.6 | 45.6 | 38.3 | 23.6 | 35.8 |
| 120 | 350 | 44.9 | 41.1 | 32.0 | 33.3 | 22.6 | 25.1 | 43.0 | 32.7 | 23.9 | 33.2 |
| | 450 | 43.6 | 43.0 | 37.0 | 32.8 | 22.7 | 26.2 | 43.3 | 34.9 | 24.5 | 34.2 |
| | 550 | 43.1 | 40.8 | 27.7 | 35.5 | 23.5 | 23.3 | 41.9 | 31.6 | 23.4 | 32.3 |
| | | YxGRxND | | | | | | YxND (4.56) | | | ND |
| 0 | | 41.8 | 44.6 | 33.1 | 34.4 | 25.7 | 25.3 | 43.2 | 33.8 | 25.5 | 34.2 |
| 80 | | 44.3 | 43.4 | 37.6 | 36.1 | 24.6 | 24.7 | 43.8 | 36.9 | 24.6 | 35.1 |
| 120 | | 43.8 | 41.6 | 32.2 | 33.9 | 22.9 | 24.9 | 42.7 | 33.1 | 23.9 | 33.2 |
| | | YxGRxSD | | | | | | YxSD | | | SD |
| | 350 | 42.3 | 42.9 | 34.5 | 35.9 | 24.9 | 25.4 | 42.6 | 35.2 | 25.1 | 34.3 |
| | 450 | 43.1 | 44.4 | 34.5 | 34.1 | 24.6 | 25.1 | 43.8 | 34.3 | 24.9 | 34.3 |
| | 550 | 44.6 | 42.3 | 34.0 | 34.4 | 23.7 | 24.4 | 43.4 | 34.2 | 24.0 | 33.9 |
| | | YxGR | | | | | | Y | | | Grand mean |
| | | 43.3 | 43.2 | 34.3 | 34.8 | 24.4 | 25.0 | 43.3 | 34.6 | 24.7 | 34.2 |

**Pn(Z59)**

| Significant effect | HSD(alfa=0.05) |
|---|---|
| Y | 8.227 |

**Table 2.8 E(Z59) (mmol($H_2O$)**

| ND | SD | 2015 | | 2016 | | 2017 | | 2015 | 2016 | 2017 | |
|---|---|---|---|---|---|---|---|---|---|---|---|
| | | R0 | R1 | R0 | R1 | R0 | R1 | | | | |
| | | YxGRxNDxSD | | | | | | YxNDxSD | | | NxSD |
| 0 | 350 | 3.90 | 5.50 | 2.00 | 3.93 | 5.16 | 4.23 | 4.70 | 2.97 | 4.70 | 4.12 |
| | 450 | 5.47 | 6.20 | 2.73 | 3.17 | 4.29 | 4.38 | 5.83 | 2.95 | 4.34 | 4.37 |
| | 550 | 5.00 | 4.80 | 3.20 | 3.67 | 4.09 | 4.14 | 4.90 | 3.43 | 4.12 | 4.15 |
| 80 | 350 | 5.53 | 5.63 | 3.50 | 3.73 | 3.80 | 3.68 | 5.58 | 3.62 | 3.74 | 4.31 |
| | 450 | 4.83 | 5.17 | 2.53 | 2.80 | 4.59 | 4.04 | 5.00 | 2.67 | 4.32 | 3.99 |
| | 550 | 5.70 | 5.23 | 4.10 | 4.03 | 3.38 | 4.38 | 5.47 | 4.07 | 3.88 | 4.47 |
| 120 | 350 | 4.50 | 4.27 | 2.67 | 3.07 | 3.37 | 3.91 | 4.38 | 2.87 | 3.64 | 3.63 |
| | 450 | 3.33 | 4.13 | 2.57 | 2.87 | 4.50 | 3.98 | 3.73 | 2.72 | 4.24 | 3.56 |
| | 550 | 4.83 | 3.90 | 2.97 | 3.90 | 4.15 | 3.72 | 4.37 | 3.43 | 3.94 | 3.91 |
| | | YxGRxND | | | | | | YxND (4.56) | | | ND |
| 0 | | 4.79 | 5.50 | 2.64 | 3.59 | 4.51 | 4.25 | 5.14 | 3.12 | 4.38 | 4.21 |
| 80 | | 5.36 | 5.34 | 3.38 | 3.52 | 3.93 | 4.03 | 5.35 | 3.45 | 3.98 | 4.26 |
| 120 | | 4.22 | 4.10 | 2.73 | 3.28 | 4.00 | 3.87 | 4.16 | 3.01 | 3.94 | 3.70 |
| | | YxGRxSD | | | | | | YxSD | | | SD |
| | 350 | 4.64 | 5.13 | 2.72 | 3.58 | 4.11 | 3.94 | 4.89 | 3.15 | 4.03 | 4.02 |
| | 450 | 4.54 | 5.17 | 2.61 | 2.94 | 4.46 | 4.13 | 4.86 | 2.78 | 4.30 | 3.98 |
| | 550 | 5.18 | 4.64 | 3.42 | 3.87 | 3.87 | 4.08 | 4.91 | 3.64 | 3.98 | 4.18 |
| | | YxGR | | | | | | Y | | | Grand mean |
| | | 4.79 | 4.98 | 2.92 | 3.46 | 4.15 | 4.05 | 4.89 | 3.19 | 4.10 | 4.06 |

**E(Z59)**

| Significant effect | HSD(alfa=0.05) |
|---|---|
| Y | 1.445 |
| Y*SD | 0.882 |

**Table 2.9 WUE(Z59)**

| ND | SD | 2015 | | 2016 | | 2017 | | 2015 | 2016 | 2017 | |
|---|---|---|---|---|---|---|---|---|---|---|---|
| | | R0 | R1 | R0 | R1 | R0 | R1 | | | | |
| | | YxGRxNDxSD | | | | | | YxNDxSD | | | NxSD |
| 0 | 350 | 10.4 | 8.3 | 21.6 | 14.2 | 5.5 | 6.7 | 9.4 | 17.9 | 6.1 | 11.1 |
| | 450 | 7.6 | 7.5 | 16.7 | 17.6 | 5.9 | 6.0 | 7.6 | 17.1 | 6.0 | 10.2 |
| | 550 | 9.1 | 9.0 | 14.0 | 10.0 | 6.7 | 6.7 | 9.0 | 12.0 | 6.7 | 9.2 |
| 80 | 350 | 8.1 | 7.8 | 10.6 | 10.4 | 6.6 | 7.2 | 7.9 | 10.5 | 6.9 | 8.5 |
| | 450 | 9.7 | 8.6 | 14.4 | 13.1 | 5.9 | 6.3 | 9.2 | 13.7 | 6.1 | 9.7 |
| | 550 | 8.3 | 8.9 | 10.3 | 9.1 | 6.8 | 5.6 | 8.6 | 9.7 | 6.2 | 8.2 |
| 120 | 350 | 10.3 | 10.1 | 13.7 | 11.2 | 7.3 | 6.5 | 10.2 | 12.5 | 6.9 | 9.8 |
| | 450 | 13.8 | 10.6 | 15.0 | 21.6 | 5.8 | 6.6 | 12.2 | 18.3 | 6.2 | 12.3 |
| | 550 | 9.1 | 11.6 | 9.4 | 9.5 | 6.2 | 6.4 | 10.4 | 9.4 | 6.3 | 8.7 |
| | | YxGRxND | | | | | | YxND (4.56) | | | ND |
| 0 | | 9.0 | 8.3 | 17.4 | 13.9 | 6.0 | 6.5 | 8.7 | 15.7 | 6.3 | 10.2 |
| 80 | | 8.7 | 8.4 | 11.8 | 10.9 | 6.5 | 6.4 | 8.6 | 11.3 | 6.4 | 8.8 |
| 120 | | 11.1 | 10.8 | 12.7 | 14.1 | 6.4 | 6.5 | 10.9 | 13.4 | 6.5 | 10.3 |
| | | YxGRxSD | | | | | | YxSD | | | SD |
| | 350 | 9.6 | 8.7 | 15.3 | 12.0 | 6.5 | 6.8 | 9.2 | 13.6 | 6.6 | 9.8 |
| | 450 | 10.4 | 8.9 | 15.4 | 17.4 | 5.9 | 6.3 | 9.7 | 16.4 | 6.1 | 10.7 |
| | 550 | 8.8 | 9.8 | 11.2 | 9.5 | 6.6 | 6.2 | 9.3 | 10.4 | 6.4 | 8.7 |
| | | YxGR | | | | | | Y | | | Grand mean |
| | | 9.6 | 9.2 | 14.0 | 13.0 | 6.3 | 6.4 | 9.4 | 13.5 | 6.4 | 9.7 |

**WUE(Z59)**

| Significant effect | HSD(alfa=0.05) |
|---|---|
| Y | 2.270 |
| Y*SD | 4.586 |

**Tables 3.1-3.8. The main and interaction effects of years and agronomic factors for the yield components and yield of *T. durum*.**

Symbols:

| | |
|---|---|
| Y | Year |
| GR | Growth regulator (0 - no, 1 - yes) |
| ND | Nitrogen dose in kg $ha^{-1}$ |
| SD | Sowing density (No of plants $m^{-2}$) |
| HSD | Honest Significant Difference acc. to Tukey's T-test |

**Table 3.1 Stem length (cm)**

| ND | SD | 2015 | | 2016 | | 2017 | | 2015 | 2016 | 2017 | |
|---|---|---|---|---|---|---|---|---|---|---|---|
| | | R0 | R1 | R0 | R1 | R0 | R1 | | | | |
| | | YxGRxNDxSD | | | | | | YxNDxSD | | | NxSD |
| 0 | 350 | 65.8 | 64.0 | 64.0 | 64.8 | 62.0 | 65.7 | 64.9 | 64.4 | 63.9 | 64.4 |
| | 450 | 65.8 | 65.4 | 58.7 | 62.2 | 61.3 | 64.3 | 65.6 | 60.5 | 62.8 | 63.0 |
| | 550 | 66.4 | 64.0 | 60.1 | 63.2 | 61.5 | 62.8 | 65.2 | 61.7 | 62.2 | 63.0 |
| 80 | 350 | 68.1 | 66.2 | 73.4 | 71.2 | 72.9 | 71.5 | 67.1 | 72.3 | 72.2 | 70.5 |
| | 450 | 69.7 | 66.7 | 71.1 | 71.9 | 71.4 | 72.7 | 68.2 | 71.5 | 72.0 | 70.6 |
| | 550 | 66.3 | 63.2 | 73.3 | 75.2 | 72.0 | 71.5 | 64.7 | 74.3 | 71.8 | 70.3 |
| 120 | 350 | 67.7 | 66.4 | 75.3 | 73.5 | 72.5 | 75.6 | 67.0 | 74.4 | 74.0 | 71.8 |
| | 450 | 68.7 | 64.8 | 72.0 | 70.2 | 73.1 | 76.3 | 66.7 | 71.1 | 74.7 | 70.8 |
| | 550 | 70.5 | 69.3 | 71.6 | 73.9 | 73.8 | 71.9 | 69.9 | 72.8 | 72.8 | 71.8 |
| | | YxGRxND | | | | | | YxND (4.56) | | | ND |
| 0 | | 66.0 | 64.5 | 60.9 | 63.4 | 61.6 | 64.3 | 65.2 | 62.2 | 62.9 | 63.4 |
| 80 | | 68.0 | 65.4 | 72.6 | 72.8 | 72.1 | 71.9 | 66.7 | 72.7 | 72.0 | 70.5 |
| 120 | | 68.9 | 66.8 | 73.0 | 72.6 | 73.1 | 74.6 | 67.9 | 72.8 | 73.8 | 71.5 |
| | | YxGRxSD | | | | | | YxSD | | | SD |
| | 350 | 67.2 | 65.5 | 70.9 | 69.8 | 69.2 | 70.9 | 66.3 | 70.4 | 70.0 | 68.9 |
| | 450 | 68.1 | 65.6 | 67.3 | 68.1 | 68.6 | 71.1 | 66.9 | 67.7 | 69.8 | 68.1 |
| | 550 | 67.7 | 65.5 | 68.3 | 70.8 | 69.1 | 68.8 | 66.6 | 69.6 | 68.9 | 68.4 |
| | | YxGR | | | | | | Y | | | Grand mean |
| | | 67.6 | 65.5 | 68.8 | 69.6 | 69.0 | 70.2 | 66.6 | 69.2 | 69.6 | 68.5 |

| Significant effect | HSD(alfa=0.05) |
|---|---|
| ND | 1.89 |
| Y*ND | 4.56 |

**Table 3.2 Ear length (cm)**

| N | SD | 2015 | | 2016 | | 2017 | | 2015 | 2016 | 2017 | |
|---|---|---|---|---|---|---|---|---|---|---|---|
| | | R0 | R1 | R0 | R1 | R0 | R1 | | | | |
| | | YxGRxNDxSD | | | | | | YxNDxSD | | | NDxSD |
| 0 | 350 | 5.20 | 5.13 | 4.80 | 4.92 | 4.89 | 5.35 | 5.17 | 4.86 | 5.12 | 5.05 |
| | 450 | 5.18 | 5.15 | 3.97 | 4.61 | 6.39 | 4.54 | 5.17 | 4.29 | 5.46 | 4.97 |
| | 550 | 4.90 | 4.88 | 4.19 | 4.73 | 4.75 | 4.62 | 4.89 | 4.46 | 4.68 | 4.68 |
| 80 | 350 | 5.58 | 5.45 | 5.77 | 5.35 | 5.64 | 5.98 | 5.52 | 5.56 | 5.81 | 5.63 |
| | 450 | 5.43 | 5.40 | 5.23 | 4.88 | 5.50 | 5.59 | 5.42 | 5.06 | 5.55 | 5.34 |
| | 550 | 5.12 | 5.17 | 5.17 | 5.38 | 5.36 | 5.24 | 5.14 | 5.28 | 5.30 | 5.24 |
| 120 | 350 | 5.45 | 5.47 | 5.59 | 5.71 | 5.93 | 5.86 | 5.46 | 5.65 | 5.89 | 5.67 |
| | 450 | 5.42 | 5.65 | 5.19 | 4.83 | 5.39 | 5.43 | 5.53 | 5.01 | 5.41 | 5.32 |
| | 550 | 70.5 | 69.3 | 71.6 | 73.9 | 73.8 | 71.9 | 69.9 | 72.8 | 72.8 | 71.8 |
| | | YxGRxND | | | | | | YxND (4.56) | | | ND |
| 0 | | 5.09 | 5.06 | 4.32 | 4.75 | 5.34 | 4.83 | 5.08 | 4.54 | 5.09 | 4.90 |
| 80 | | 5.38 | 5.34 | 5.39 | 5.20 | 5.50 | 5.61 | 5.36 | 5.30 | 5.55 | 5.40 |
| 120 | | 5.30 | 5.51 | 5.29 | 5.33 | 5.59 | 5.56 | 5.41 | 5.31 | 5.57 | 5.43 |
| | | YxGRxSD | | | | | | YxSD | | | SD |
| | 350 | 5.41 | 5.35 | 5.39 | 5.32 | 5.49 | 5.73 | 5.38 | 5.36 | 5.61 | 5.45 |
| | 450 | 5.34 | 5.40 | 4.80 | 4.77 | 5.76 | 5.19 | 5.37 | 4.79 | 5.47 | 5.21 |
| | 550 | 5.02 | 5.16 | 4.82 | 5.19 | 5.18 | 5.08 | 5.09 | 5.00 | 5.13 | 5.07 |
| | | YxGR | | | | | | Y | | | Grand mean |
| | | 5.26 | 5.30 | 5.00 | 5.09 | 5.48 | 5.33 | 5.28 | 5.05 | 5.40 | 5.24 |

| Significant effect | HSD(alfa=0.05) |
|---|---|
| Y | 0.253 |
| ND | 0.248 |
| SD | 0.232 |

**Table 3.3 Kernels per ear**

| N | SD | 2015 | | 2016 | | 2017 | | 2015 | 2016 | 2017 | |
|---|---|---|---|---|---|---|---|---|---|---|---|
| | | R0 | R1 | R0 | R1 | R0 | R1 | | | | |
| | | YxGRxNDxSD | | | | | | YxNDxSD | | | NDxSD |
| 0 | 350 | 25.13 | 26.20 | 20.83 | 23.70 | 19.73 | 25.80 | 25.67 | 22.27 | 22.77 | 23.57 |
| | 450 | 26.53 | 27.70 | 19.07 | 21.60 | 18.63 | 21.93 | 27.12 | 20.33 | 20.28 | 22.58 |
| | 550 | 25.00 | 23.33 | 18.73 | 22.67 | 17.07 | 20.57 | 24.17 | 20.70 | 18.82 | 21.23 |
| 80 | 350 | 27.13 | 28.33 | 28.07 | 26.17 | 19.27 | 26.67 | 27.73 | 27.12 | 22.97 | 25.94 |
| | 450 | 27.07 | 27.47 | 23.83 | 23.83 | 23.50 | 21.30 | 27.27 | 23.83 | 22.40 | 24.50 |
| | 550 | 24.47 | 21.50 | 24.13 | 25.13 | 26.83 | 21.70 | 22.98 | 24.63 | 24.27 | 23.96 |
| 120 | 350 | 27.17 | 26.23 | 31.43 | 31.67 | 23.50 | 28.90 | 26.70 | 31.55 | 26.20 | 28.15 |
| | 450 | 26.77 | 26.40 | 25.47 | 24.77 | 21.10 | 25.27 | 26.58 | 25.12 | 23.18 | 24.96 |
| | 550 | 27.10 | 24.80 | 24.90 | 26.87 | 21.73 | 25.53 | 25.95 | 25.88 | 23.63 | 25.16 |
| | | YxGRxND | | | | | | YxND (4.56) | | | ND |
| 0 | | 25.56 | 25.74 | 19.54 | 22.66 | 18.48 | 22.77 | 25.65 | 21.10 | 20.62 | 22.46 |
| 80 | | 26.22 | 25.77 | 25.34 | 25.04 | 23.20 | 23.22 | 25.99 | 25.19 | 23.21 | 24.80 |
| 120 | | 27.01 | 25.81 | 27.27 | 27.77 | 22.11 | 26.57 | 26.41 | 27.52 | 24.34 | 26.09 |
| | | YxGRxSD | | | | | | YxSD | | | SD |
| | 350 | 26.48 | 26.92 | 26.78 | 27.18 | 20.83 | 27.12 | 26.70 | 26.98 | 23.98 | 25.89 |
| | 450 | 26.79 | 27.19 | 22.79 | 23.40 | 21.08 | 22.83 | 26.99 | 23.09 | 21.96 | 24.01 |
| | 550 | 25.52 | 23.21 | 22.59 | 24.89 | 21.88 | 22.60 | 24.37 | 23.74 | 22.24 | 23.45 |
| | | YxGR | | | | | | Y | | | Grand mean |
| | | 26.26 | 25.77 | 24.05 | 25.16 | 21.26 | 24.19 | 26.02 | 24.60 | 22.72 | 24.45 |

| Significant effect | HSD(alfa=0.05) |
|---|---|
| ND | 2.76 |
| GS | 1.46 |

**Table 3.4 Kernels weight (g)**

| N | SD | 2015 | | 2016 | | 2017 | | 2015 | 2016 | 2017 | |
|---|---|---|---|---|---|---|---|---|---|---|---|
| | | R0 | R1 | R0 | R1 | R0 | R1 | | | | |
| | | YxGRxNDxSD | | | | | | YxNDxSD | | | NDxSD |
| 0 | 350 | 1.50 | 1.49 | 1.05 | 1.15 | 0.83 | 1.02 | 1.50 | 1.10 | 0.93 | 1.17 |
| | 450 | 1.50 | 1.61 | 0.89 | 1.02 | 0.81 | 0.87 | 1.55 | 0.95 | 0.84 | 1.12 |
| | 550 | 1.40 | 1.28 | 0.80 | 1.05 | 0.68 | 0.72 | 1.34 | 0.93 | 0.70 | 0.99 |
| 80 | 350 | 1.50 | 1.58 | 1.36 | 1.27 | 0.69 | 0.93 | 1.54 | 1.31 | 0.81 | 1.22 |
| | 450 | 1.53 | 1.49 | 1.16 | 1.10 | 0.94 | 0.94 | 1.51 | 1.13 | 0.94 | 1.19 |
| | 550 | 1.34 | 1.08 | 1.13 | 1.18 | 1.02 | 0.88 | 1.21 | 1.15 | 0.95 | 1.10 |
| 120 | 350 | 1.55 | 1.38 | 1.53 | 1.48 | 0.91 | 1.08 | 1.47 | 1.51 | 0.99 | 1.32 |
| | 450 | 1.48 | 1.43 | 1.18 | 1.20 | 0.83 | 1.02 | 1.46 | 1.19 | 0.93 | 1.19 |
| | 550 | 1.49 | 1.25 | 1.17 | 1.25 | 0.94 | 0.95 | 1.37 | 1.21 | 0.95 | 1.18 |
| | | YxGRxND | | | | | | YxND (4.56) | | | ND |
| 0 | | 1.47 | 1.46 | 0.91 | 1.07 | 0.77 | 0.87 | 1.46 | 0.99 | 0.82 | 1.09 |
| 80 | | 1.46 | 1.38 | 1.22 | 1.18 | 0.89 | 0.92 | 1.42 | 1.20 | 0.90 | 1.17 |
| 120 | | 1.51 | 1.36 | 1.29 | 1.31 | 0.89 | 1.02 | 1.43 | 1.30 | 0.96 | 1.23 |
| | | YxGRxSD | | | | | | YxSD | | | SD |
| | 350 | 1.52 | 1.49 | 1.31 | 1.30 | 0.81 | 1.01 | 1.50 | 1.31 | 0.91 | 1.24 |
| | 450 | 1.50 | 1.51 | 1.08 | 1.11 | 0.86 | 0.94 | 1.51 | 1.09 | 0.90 | 1.17 |
| | 550 | 1.41 | 1.20 | 1.04 | 1.16 | 0.88 | 0.85 | 1.31 | 1.10 | 0.87 | 1.09 |
| | | YxGR | | | | | | Y | | | Grand mean |
| | | 1.48 | 1.40 | 1.14 | 1.19 | 0.85 | 0.93 | 1.44 | 1.17 | 0.89 | 1.17 |

| Significant effect | HSD(alfa=0.05) |
|---|---|
| Y | 0.184 |
| ND | 0.128 |
| Y*ND | 0.309 |
| SD | 0.079 |
| Y*SD | 0.183 |

**Table 3.5 Grain weight (t $ha^{-1}$)**

| N | SD | 2015 | | 2016 | | 2017 | | 2015 | 2016 | 2017 | |
|---|---|---|---|---|---|---|---|---|---|---|---|
| | | R0 | R1 | R0 | R1 | R0 | R1 | | | | |
| | | YxGRxNDxSD | | | | | | YxNDxSD | | | NDxSD |
| 0 | 350 | 4.97 | 4.37 | 4.06 | 4.01 | 2.82 | 2.78 | 4.67 | 4.03 | 2.80 | 3.83 |
| | 450 | 5.17 | 4.74 | 4.11 | 4.23 | 2.75 | 2.70 | 4.95 | 4.17 | 2.73 | 3.95 |
| | 550 | 5.03 | 4.51 | 3.97 | 4.04 | 2.83 | 2.75 | 4.77 | 4.00 | 2.79 | 3.86 |
| 80 | 350 | 5.17 | 4.70 | 5.56 | 5.36 | 4.59 | 4.50 | 4.94 | 5.46 | 4.54 | 4.98 |
| | 450 | 5.32 | 4.68 | 5.79 | 5.58 | 4.49 | 4.56 | 5.00 | 5.69 | 4.52 | 5.07 |
| | 550 | 4.83 | 4.73 | 5.98 | 5.86 | 4.63 | 4.75 | 4.78 | 5.92 | 4.69 | 5.13 |
| 120 | 350 | 4.74 | 4.97 | 6.54 | 6.61 | 4.17 | 3.99 | 4.85 | 6.57 | 4.08 | 5.17 |
| | 450 | 4.99 | 5.39 | 6.05 | 5.96 | 4.26 | 4.22 | 5.19 | 6.01 | 4.24 | 5.15 |
| | 550 | 5.30 | 5.31 | 6.52 | 6.70 | 4.50 | 4.45 | 5.30 | 6.61 | 4.47 | 5.46 |
| | | YxGRxND | | | | | | YxND (4.56) | | | ND |
| 0 | | 5.06 | 4.54 | 4.04 | 4.09 | 2.80 | 2.75 | 4.80 | 4.07 | 2.77 | 3.88 |
| 80 | | 5.11 | 4.70 | 5.78 | 5.60 | 4.57 | 4.60 | 4.91 | 5.69 | 4.59 | 5.06 |
| 120 | | 5.01 | 5.22 | 6.37 | 6.42 | 4.31 | 4.22 | 5.11 | 6.40 | 4.27 | 5.26 |
| | | YxGRxSD | | | | | | YxSD | | | SD |
| | 350 | 4.96 | 4.68 | 5.39 | 5.33 | 3.86 | 3.76 | 4.82 | 5.36 | 3.81 | 4.66 |
| | 450 | 5.16 | 4.93 | 5.32 | 5.26 | 3.83 | 3.83 | 5.05 | 5.29 | 3.83 | 4.72 |
| | 550 | 5.05 | 4.85 | 5.49 | 5.53 | 3.99 | 3.98 | 4.95 | 5.51 | 3.99 | 4.82 |
| | | YxGR | | | | | | Y | | | Grand mean |
| | | 5.06 | 4.82 | 5.40 | 5.37 | 3.89 | 3.86 | 4.94 | 5.38 | 3.87 | 4.73 |

| Significant effect | HSD(alfa=0.05) |
|---|---|
| Y | 1.40 |
| ND | 0.30 |
| Y*ND | 0.73 |
| SD | 0.14 |
| Y*ND*SD | 0.69 |

**Table 3.6 Straw weight (t ha$^{-1}$)**

| N | SD | 2015 | | 2016 | | 2017 | | 2015 | 2016 | 2017 | |
|---|---|---|---|---|---|---|---|---|---|---|---|
| | | R0 | R1 | R0 | R1 | R0 | R1 | | | | |
| | | YxGRxNDxSD | | | | | | YxNDxSD | | | NDxSD |
| 0 | 350 | 2.29 | 2.29 | 2.29 | 2.25 | 1.64 | 1.75 | 2.29 | 2.27 | 1.70 | 2.08 |
| | 450 | 2.29 | 2.67 | 2.51 | 2.48 | 1.52 | 1.79 | 2.48 | 2.50 | 1.66 | 2.21 |
| | 550 | 2.29 | 2.48 | 2.40 | 2.29 | 1.94 | 1.83 | 2.38 | 2.34 | 1.89 | 2.20 |
| 80 | 350 | 2.67 | 2.48 | 2.78 | 2.51 | 2.86 | 2.59 | 2.57 | 2.65 | 2.72 | 2.65 |
| | 450 | 2.67 | 2.29 | 3.54 | 2.51 | 2.44 | 2.59 | 2.48 | 3.03 | 2.51 | 2.67 |
| | 550 | 2.29 | 2.29 | 3.09 | 2.97 | 2.93 | 3.09 | 2.29 | 3.03 | 3.01 | 2.77 |
| 120 | 350 | 2.48 | 2.48 | 3.54 | 3.28 | 3.05 | 3.16 | 2.48 | 3.41 | 3.10 | 3.00 |
| | 450 | 2.29 | 2.86 | 3.28 | 2.86 | 2.70 | 2.74 | 2.57 | 3.07 | 2.72 | 2.79 |
| | 550 | 2.29 | 3.05 | 3.35 | 3.47 | 3.01 | 2.90 | 2.67 | 3.41 | 2.95 | 3.01 |
| | | YxGRxND | | | | | | YxND (4.56) | | | ND |
| 0 | | 2.29 | 2.48 | 2.40 | 2.34 | 1.70 | 1.79 | 2.38 | 2.37 | 1.75 | 2.17 |
| 80 | | 2.54 | 2.35 | 3.14 | 2.67 | 2.74 | 2.76 | 2.44 | 2.90 | 2.75 | 2.70 |
| 120 | | 2.35 | 2.79 | 3.39 | 3.20 | 2.92 | 2.93 | 2.57 | 3.30 | 2.93 | 2.93 |
| | | YxGRxSD | | | | | | YxSD | | | SD |
| | 350 | 2.48 | 2.41 | 2.87 | 2.68 | 2.51 | 2.50 | 2.44 | 2.77 | 2.51 | 2.58 |
| | 450 | 2.41 | 2.60 | 3.11 | 2.62 | 2.22 | 2.37 | 2.51 | 2.86 | 2.30 | 2.56 |
| | 550 | 2.29 | 2.60 | 2.95 | 2.91 | 2.63 | 2.60 | 2.44 | 2.93 | 2.62 | 2.66 |
| | | YxGR | | | | | | Y | | | Grand mean |
| | | 2.39 | 2.54 | 2.98 | 2.73 | 2.46 | 2.49 | 2.47 | 2.86 | 2.47 | 2.60 |

| Significant effect | HSD(alfa=0.05) |
|---|---|
| ND | 0.222 |
| Y*ND | 0.536 |

**Table 3.7 Harvest index**

| N | SD | 2015 | | 2016 | | 2017 | | 2015 | 2016 | 2017 | |
|---|---|---|---|---|---|---|---|---|---|---|---|
| | | R0 | R1 | R0 | R1 | R0 | R1 | | | | |
| | | YxGRxNDxSD | | | | | | YxNDxSD | | | NDxSD |
| 0 | 350 | 0.68 | 0.66 | 0.64 | 0.64 | 0.63 | 0.61 | 0.67 | 0.64 | 0.62 | 0.64 |
| | 450 | 0.69 | 0.64 | 0.62 | 0.63 | 0.64 | 0.60 | 0.67 | 0.63 | 0.62 | 0.64 |
| | 550 | 0.68 | 0.65 | 0.62 | 0.64 | 0.59 | 0.60 | 0.67 | 0.63 | 0.60 | 0.63 |
| 80 | 350 | 0.66 | 0.65 | 0.67 | 0.69 | 0.62 | 0.64 | 0.65 | 0.68 | 0.63 | 0.65 |
| | 450 | 0.66 | 0.68 | 0.62 | 0.69 | 0.65 | 0.64 | 0.67 | 0.66 | 0.64 | 0.66 |
| | 550 | 0.68 | 0.67 | 0.66 | 0.66 | 0.61 | 0.61 | 0.68 | 0.66 | 0.61 | 0.65 |
| 120 | 350 | 0.66 | 0.67 | 0.65 | 0.67 | 0.58 | 0.56 | 0.66 | 0.66 | 0.57 | 0.63 |
| | 450 | 0.69 | 0.65 | 0.65 | 0.68 | 0.61 | 0.61 | 0.67 | 0.66 | 0.61 | 0.65 |
| | 550 | 0.69 | 0.63 | 0.66 | 0.66 | 0.60 | 0.61 | 0.66 | 0.66 | 0.60 | 0.64 |
| | | YxGRxND | | | | | | YxND (4.56) | | | ND |
| 0 | | 0.69 | 0.65 | 0.63 | 0.64 | 0.62 | 0.61 | 0.67 | 0.63 | 0.61 | 0.64 |
| 80 | | 0.67 | 0.67 | 0.65 | 0.68 | 0.63 | 0.63 | 0.67 | 0.67 | 0.63 | 0.65 |
| 120 | | 0.68 | 0.65 | 0.65 | 0.67 | 0.60 | 0.59 | 0.67 | 0.66 | 0.59 | 0.64 |
| | | YxGRxSD | | | | | | YxSD | | | SD |
| | 350 | 0.67 | 0.66 | 0.65 | 0.66 | 0.61 | 0.60 | 0.66 | 0.66 | 0.61 | 0.64 |
| | 450 | 0.68 | 0.66 | 0.63 | 0.67 | 0.63 | 0.62 | 0.67 | 0.65 | 0.63 | 0.65 |
| | 550 | 0.69 | 0.65 | 0.65 | 0.65 | 0.60 | 0.61 | 0.67 | 0.65 | 0.60 | 0.64 |
| | | YxGR | | | | | | Y | | | Grand mean |
| | | 0.68 | 0.66 | 0.64 | 0.66 | 0.62 | 0.61 | 0.67 | 0.65 | 0.61 | 0.64 |

| Significant effect | HSD(alfa=0.05) |
|---|---|
| Y | 0.0335 |

**Table 3.8 Biological yield (t ha$^{-1}$)**

| N | SD | 2015 R0 | 2015 R1 | 2016 R0 | 2016 R1 | 2017 R0 | 2017 R1 | 2015 | 2016 | 2017 | |
|---|---|---|---|---|---|---|---|---|---|---|---|
| | | YxGRxNDxSD | | | | | | YxNDxSD | | | NDxSD |
| 0 | 350 | 7.25 | 6.65 | 6.34 | 6.26 | 4.46 | 4.54 | 6.95 | 6.30 | 4.50 | 5.92 |
| | 450 | 7.45 | 7.40 | 6.62 | 6.70 | 4.27 | 4.50 | 7.43 | 6.66 | 4.38 | 6.16 |
| | 550 | 7.32 | 6.99 | 6.37 | 6.33 | 4.77 | 4.58 | 7.15 | 6.35 | 4.68 | 6.06 |
| 80 | 350 | 7.84 | 7.18 | 8.34 | 7.88 | 7.45 | 7.09 | 7.51 | 8.11 | 7.27 | 7.63 |
| | 450 | 7.99 | 6.97 | 9.33 | 8.10 | 6.93 | 7.15 | 7.48 | 8.71 | 7.04 | 7.74 |
| | 550 | 7.11 | 7.01 | 9.07 | 8.83 | 7.57 | 7.84 | 7.06 | 8.95 | 7.70 | 7.90 |
| 120 | 350 | 7.22 | 7.44 | 10.08 | 9.88 | 7.22 | 7.15 | 7.33 | 9.98 | 7.18 | 8.17 |
| | 450 | 7.28 | 8.24 | 9.33 | 8.82 | 6.97 | 6.96 | 7.76 | 9.07 | 6.97 | 7.93 |
| | 550 | 7.58 | 8.35 | 9.87 | 10.16 | 7.51 | 7.34 | 7.97 | 10.02 | 7.43 | 8.47 |
| | | YxGRxND | | | | | | YxND (4.56) | | | ND |
| 0 | | 7.34 | 7.01 | 6.44 | 6.43 | 4.50 | 4.54 | 7.18 | 6.44 | 4.52 | 6.04 |
| 80 | | 7.65 | 7.05 | 8.91 | 8.27 | 7.31 | 7.36 | 7.35 | 8.59 | 7.34 | 7.76 |
| 120 | | 7.36 | 8.01 | 9.76 | 9.62 | 7.23 | 7.15 | 7.69 | 9.69 | 7.19 | 8.19 |
| | | YxGRxSD | | | | | | YxSD | | | SD |
| | 350 | 7.44 | 7.09 | 8.26 | 8.01 | 6.37 | 6.26 | 7.26 | 8.13 | 6.32 | 7.24 |
| | 450 | 7.57 | 7.54 | 8.43 | 7.87 | 6.05 | 6.20 | 7.55 | 8.15 | 6.13 | 7.28 |
| | 550 | 7.34 | 7.45 | 8.44 | 8.44 | 6.61 | 6.59 | 7.40 | 8.44 | 6.60 | 7.48 |
| | | YxGR | | | | | | Y | | | Grand mean |
| | | 7.45 | 7.36 | 8.37 | 8.11 | 6.35 | 6.35 | 7.40 | 8.24 | 6.35 | 7.33 |

| Significant effect | HSD(alfa=0.05) |
|---|---|
| Y | 1.859 |
| ND | 0.421 |
| Y x ND | 1.015 |

**Table 4. Outer weights and loadings of manifest variables.**

| Latent variable* | Manifest variable | Outer weights, $w_i$ | Loadings, $l_i$ |
|---|---|---|---|
| A | GR | -0.007 | -0.007 |
| | ND | 0.781 | 0.781 |
| | SD | 0.624 | 0.624 |
| CC32 | Da32 | 0.201 | 0.771 |
| | Te32 | 0.350 | 0.920 |
| | Pr32 | 0.587 | 0.892 |
| PP32 | P32 | 0.454 | 0.951 |
| | E32 | 0.585 | 0.971 |
| BP32 | L32 | 0.728 | 0.881 |
| | S32 | 0.497 | 0.721 |
| CC32 | Da45 | -0.412 | -0.716 |
| | Te45 | -0.566 | -0.833 |
| | Pr45 | 0.645 | 0.363 |
| PP45 | P45 | 0.547 | 0.921 |
| | E45 | 0.540 | 0.919 |
| BP45 | L32 | -0.528 | -0.711 |
| | S45 | 0.727 | 0.860 |
| CC32 | Da59 | 0.353 | 0.978 |
| | Te59 | 0.324 | 0.988 |
| | Pr59 | 0.335 | 0.997 |
| PP59 | P59 | -0.560 | -0.868 |
| | E59 | 0.584 | 0.879 |
| BP59 | L59 | 0.508 | 0.648 |
| | S59 | 0.775 | 0.866 |
| YC | SL | 0.364 | 0.976 |
| | EL | 0.364 | 0.976 |
| | NK | -0.329 | -0.876 |
| | KW | 0.324 | 0.657 |
| CC | DAYS | 0.197 | 0.629 |
| | TEMP | 0.381 | 0.943 |
| | PREC | 0.396 | 0.768 |
| BY | GRAIN | 0.614 | 0.959 |
| | STRAW | 0.446 | 0.922 |

* Refer to Table 4 of the paper for the legend
